# Loss of the cleaved-protamine 2 domain leads to incomplete histone-to-protamine exchange and infertility in mice

**DOI:** 10.1371/journal.pgen.1010272

**Published:** 2022-06-28

**Authors:** Lena Arévalo, Gina Esther Merges, Simon Schneider, Franka Enow Oben, Isabelle Sophie Neumann, Hubert Schorle

**Affiliations:** Department of Developmental Pathology, Institute of Pathology, University Hospital Bonn, Bonn, Germany; Cornell University, UNITED STATES

## Abstract

Protamines are unique sperm-specific proteins that package and protect paternal chromatin until fertilization. A subset of mammalian species expresses two protamines (PRM1 and PRM2), while in others PRM1 is sufficient for sperm chromatin packaging. Alterations of the species-specific ratio between PRM1 and PRM2 are associated with infertility. Unlike PRM1, PRM2 is generated as a precursor protein consisting of a highly conserved N-terminal domain, termed cleaved PRM2 (cP2), which is consecutively trimmed off during chromatin condensation. The carboxyterminal part, called mature PRM2 (mP2), interacts with DNA and together with PRM1, mediates chromatin-hypercondensation. The removal of the cP2 domain is believed to be imperative for proper chromatin condensation, yet, the role of cP2 is not yet understood. We generated mice lacking the cP2 domain while the mP2 is still expressed. We show that the cP2 domain is indispensable for complete sperm chromatin protamination and male mouse fertility. cP2 deficient sperm show incomplete protamine incorporation and a severely altered protamine ratio, retention of transition proteins and aberrant retention of the testis specific histone variant H2A.L.2. During epididymal transit, cP2 deficient sperm seem to undergo ROS mediated degradation leading to complete DNA fragmentation. The cP2 domain therefore seems to be a key aspect in the complex crosstalk between histones, transition proteins and protamines during sperm chromatin condensation. Overall, we present the first step towards understanding the role of the cP2 domain in paternal chromatin packaging and open up avenues for further research.

## Author summary

Protamines are small sperm-specific proteins crucial to packaging and protecting the paternal genome on its way to the fertilization site. Most mammalian species express only protamine 1. However, primates and rodents additionally express protamine 2. Protamine 2 differs mainly in its N-terminal domain (cP2), which is sequentially cleaved off during paternal chromatin packaging. Alteration in this process has been associated with infertility. However, the precise role of cP2 is still a mystery. We generated cP2 deficient mice and demonstrate, that loss of cP2 results in incomplete histone-to-protamine transition, resulting in sperm DNA degradation and infertility. Evidently, cP2 helps in orchestrating the fine-tuned dynamics of DNA-hypercondensation while protecting DNA integrity and aiding removal of DNA-bound transition proteins.

## Introduction

Chromatin structure and dynamics in the sperm nucleus are as unique as the sperm cell itself, and are of major importance to sperm function, fertilizing ability, and embryo survival. Paternal DNA is particularly vulnerable to damage, especially to oxidative stress during epididymal sperm maturation and migration, leading to a higher requirement for protection [1]. At the same time, the size and shape of the sperm cell nucleus needs to be optimized for efficient sperm movement through the female reproductive tract [2]. This is achieved by the complete reorganization of paternal chromatin from nucleo-histone to nucleo-protamine during the final steps of spermatogenesis [3,4].

Even though many studies have demonstrated that the correct execution of this transition is imperative for male fertility and embryo survival [5], surprisingly little is known about the process itself. Recent studies have only just started to unravel its molecular basis [6–8]. Barral et al. [8] were able to show that the testis specific histone variant H2A.L.2 together with TH2B and transition proteins (TNP1 and TNP2) mediates structural changes in chromatin allowing protamines to bind DNA. Protamines are small, arginine-rich proteins. Their high arginine content allows them to bind DNA with high affinity and to shield the charges of the DNA backbone more efficiently than histones [9,10]. Two types of protamines have been identified in mammals: protamine 1 (*PRM1*, PRM1) and protamine 2 (*PRM2*, PRM2). While PRM1 is a major sperm protamine found across mammals, PRM2 is only detected in the sperm of primates, most rodents, and a subset of other placental mammals [11,12]. The coding regions of *PRM1* and *PRM2* are tightly clustered and map to a small section of chromosome 16. It is highly likely that *PRM2* is the result of a *PRM1* duplication event [13,14].

Unlike PRM1, PRM2 is transcribed as a precursor. The N-terminal region of the translated PRM2, termed cleaved-PRM2 (cP2), is successively cleaved over several days while chromatin condensation is taking place. After this, only mature-PRM2 (mP2) remains bound to the completely condensed DNA [3,12,15,16]. Perturbations of PRM2 processing has been shown to lead to decreased DNA integrity and sperm dysfunction [17,18]. In previous comparative evolutionary studies, it was shown that the cP2 coding sequence is conserved in both primates and rodents. mP2, however, evolves under less selective constraint [14,19]. Changes in coding sequences of cP2 were associated with differences in sperm head size in mouse species. This association was specific to the cP2 domain and not found for mP2 [20]. A potential reason for this pattern is that changes in cP2 are selected against conserving a crucial function for reproduction, while mP2 is free to evolve under less constraint due to its proposed functional redundancy to PRM1 [14,19].

Proper PRM2 cleaving therefore seems to be crucial for successful reproduction, yet, the function of the cP2 domain and PRM2 processing are unknown to date. Establishment and analysis of PRM2 deficient mice revealed that Prm2^-/-^ males were infertile, while Prm2^+/-^ males remained fertile [6]. Of note, mice deficient for TNP1, TNP2 and H2A.L.2 show incomplete PRM2 processing (PRM2 precursor detected in mature sperm nuclei) [8,21–23].

Given that cP2 cleaving is taking place during DNA condensation in late spermiogenesis, the strong evolutionary conservation of this domain and the effect of incomplete PRM2 processing on sperm function and fertility, cP2 is likely to play an important role in the correct execution of chromatin condensation. We therefore studied the involvement of cP2 in chromatin condensation during spermiogenesis by generating and analyzing a mouse line bearing a deletion of cP2, while maintaining mP2 expression. We analyzed fertility, testis and sperm morphology, chromatin integrity and nuclear protein content of these mice and revealed that Prm2^+/Δc^ mice are already infertile and that cP2 seems necessary for complete protamination of sperm chromatin.

## Material and methods

### Ethics statement

All animal experiments were conducted according to German law of animal protection and in agreement with the approval of the local institutional animal care committees (Landesamt für Natur, Umwelt und Verbraucherschutz, North Rhine-Westphalia, Germany, AZ81-02.04.2018.A369).

### Animals

For the generation of mouse lines using CRISPR/Cas9 the F1 generation of mouse strains C57Bl/6 and DBA2 (B6D2F1) were used. Founder animals were backcrossed to C57Bl/6. Mice were maintained under standard laboratory conditions in environmentally controlled rooms (20–24°C) on a 12L:12D photoperiod with nesting material and ad libitum food and water.

### Gene-edited mouse lines

Guide RNA (gRNA) sequence pre-selection was performed using the algorithm published by [24]. Two gRNA sequences were selected based on quality scores in each case maximizing specificity and minimizing off-target action (score > 50) (S1 Table). The designed guide sequences were ordered as crisprRNA sequences (crRNA) (IDT, Leuven, Belgium) and annealed to tracrRNA (IDT) by incubating 5min at 95°C for a final concentration of 50mM of gRNA (crRNA+tracrRNA). The target site of the designed gRNAs is shown in S1A Fig. The repair template used for homology directed repair (HDR), here single-stranded oligodeoxynucleotides (ssODNs) (IDT) [25,26], are shown in S1B Fig.

Ribonucleoprotein (RNP) complexes were assembled immediately prior to delivery by incubation of 4pmol/μl Cas9 (IDT) protein, 4pmol/μl of each gRNA and 10pmol/μl ssODN in Opti-MEM medium (Thermo Fisher Scientific, Waltham, USA) for 10min at room temperature.

**Fig 1:**
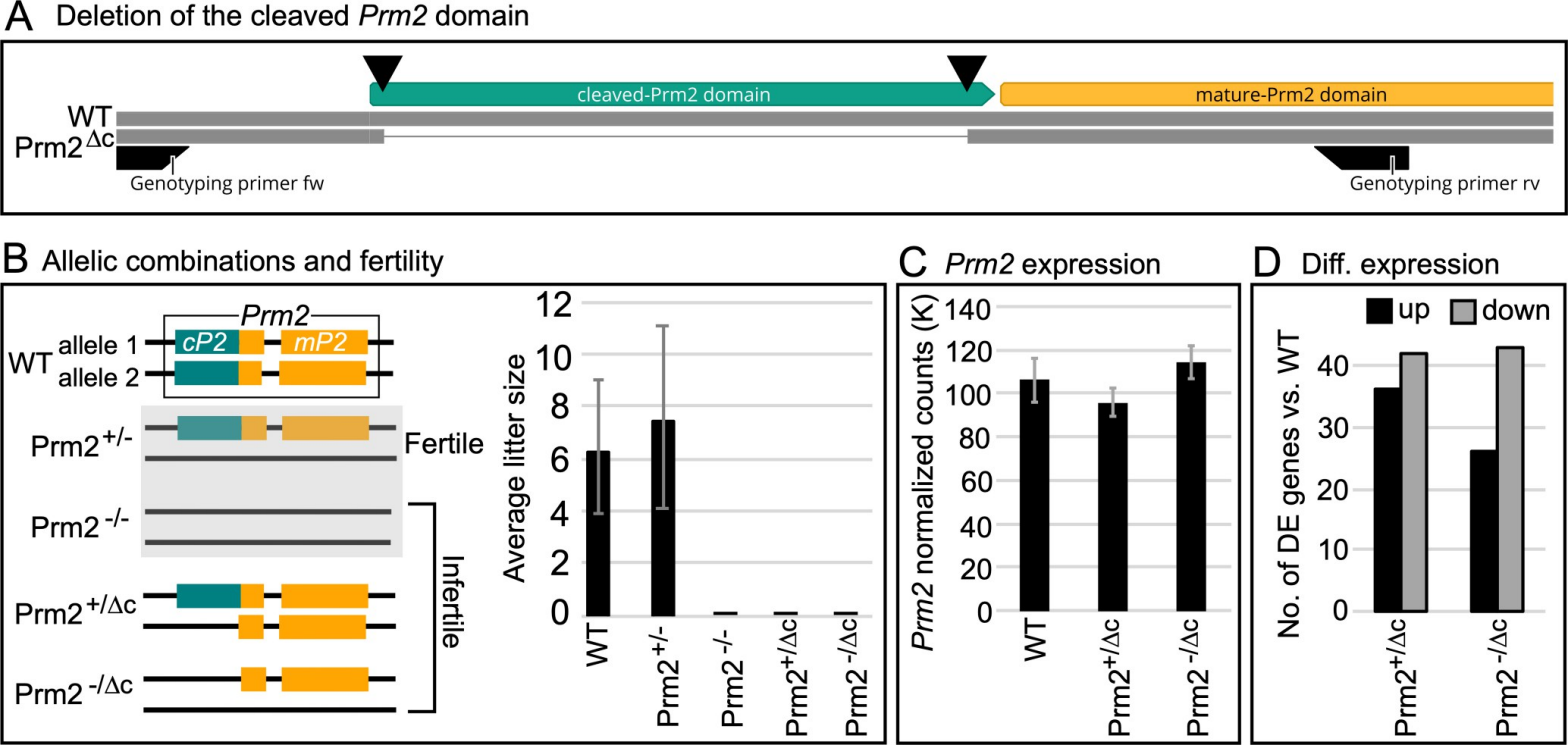
Gene editing, fertility and expression. (A) Schematic representation of the generation of cP2 deletion. Double strand breaks induced by Cas9 indicated by black triangles. (B) Schematic overview of analyzed genotypes and fertility. Prm2^+/-^ and Prm2^-/-^ [6] were included as a comparison. Bar plot of average litter size for WT, Prm2^+/-^, Prm2^-/-^, Prm2^+/Δc^ and Prm2^-/Δc^, n = 5 for each genotype. (C) Bar plot showing average DESeq2 normalized read counts of *Prm2* for WT, Prm2^+/Δc^ and Prm2^-/Δc^. (D) Bar plot showing comparison between number of differentially higher and lower expressed genes for Prm2^+/Δc^ and Prm2^-/Δc^ compared to wildtype.

To generate gene-edited founder animals, B6D2F1 females were hormonally superovulated and mated as described [6]. Oocytes were isolated from the oviducts, washed and transferred into droplets of Opti-MEM medium containing the previously prepared RNP complex and electroporated using a BioRad Gene Pulser (BioRad, Feldkirchen, Germany) (two 3ms square wave pulses at 30V with an 100ms interval). Oocytes were recovered and washed 5x in M2 medium (Merck Millipore, Darmstadt, Germany) followed by 3 washes in KSOM (Merck Millipore) medium droplets. Oocytes were then incubated in KSOM medium covered in paraffin oil overnight at 37°C. Developing 2-cell stage embryos were then transferred into the oviducts of pseudo-pregnant foster mice. Offspring was genotyped (primers, see Fig 1A and S1 Table) and positive founder animals backcrossed to C57Bl/6J for at least 3 generations before analysis. Male mice between 10 and 13 weeks of age were used for analysis.

### Fertility analysis

Five males per genotype were mated with C57Bl/6J females 1:2 and females were checked daily for the presence of a vaginal plug until at least 5 plugs per male were observed. Pregnancy rate and litter size were noted.

### Sampling and mature sperm isolation

The testes were dissected and weighed. For paraffin sectioning, testes and epididymides were fixed in either Bouin’s solution for histology or in 4% Paraformaldehyde solution for immunohistochemistry (IHC). For tubule preparations testes were transferred to PBS and tubules dissected as described in Kotaja et al. [27]. Briefly, tubules were separated and elongating and condensed spermatid containing sections were identified through their light absorption pattern using a dissection microscope. Tubule sections were squashed on a slide, frozen in liquid nitrogen for 20 seconds, fixed in 90% ethanol for 5 minutes and air dried. Tubule preparations were used for IHC. To obtain sperm samples, caudae epididymes were dissected and transferred to preheated (36–37°C) M2 medium, incised several times, squeezed with tweezers several times during a 30min incubation period at 36–37°C to ensure flushing out the whole sperm population including immotile sperm.

### Histology

Bouin-fixed testes and epididymes were paraffinized, embedded in paraffin blocks and sectioned at 3 microns. Sections were deparaffinized and stained with the Periodic acid–Schiff (PAS) procedure. Stained sections were imaged under bright field at 20x and 63x magnification using a Leica DM5500 B microscope (Leica Microsystems, Wetzlar, Germany).

### Basic sperm analysis

Isolated spermatozoa were counted for 6–12 animals per genotype using a hemocytometer. Sperm motility was analyzed for 3–4 animals per genotype by taking 5–10, 3 second video clips per animal using a Basler acA1920-155ucMED camera (Basler AG, Ahrensburg, Germany). A minimum of 200 sperm per individual were analyzed and the percentage of motile sperm calculated. Sperm viability was analyzed by eosin-nigrosin staining for 3–4 animals per genotype. Approximately 1 x 10^6^ sperm were mixed with 50μl of eosin-nigrosin dye, incubated for 30 seconds, spread on slides, air-dried and cover-slipped. A minimum of 200 spermatozoa were analyzed under bright field (Leica DMIRB microscope) and the percentage of viable spermatozoa calculated.

### RNAseq and differential expression analysis

RNA was extracted from testes after removal of the tunica albuginea using the RNeasy kit (Qiagen, Hilden, Germany). RNA integrity (RIN) was determined using the RNA Nano 6000 Assay Kit with the Agilent Bioanalyzer 2100 system (Agilent Technologies, Santa Clara, CA, USA). RIN values ranged from 7.3–10 for all samples. RNA sample quality control and library preparation were performed by the University of Bonn Core facility for Next Generation Sequencing (NGS), using the QuantSeq 3’-mRNA Library Prep (Lexogen, Greenland, NH, USA). RNAseq was performed by the University of Bonn Core facility for Next Generation Sequencing (NGS) on the Illumina HiSeq 2500 V4 platform, producing >10 million, 50bp 3’-end reads per sample.

The samples were then mapped to the mouse genome (GRCm38.89) using HISAT2 2.1 [28]. StringTie 1.3.3 [29] was used for transcript quantification and annotation. Gene annotation was retrieved from the Ensembl FTP server (ftp://ftp.ensembl.org)(GRCm38.89). The python script (preDE.py) included in the StringTie package was used to prepare DEseq2-compatible gene-level count matrices for analysis of differential gene expression. Mapping to the *Prm2* genomic location was visualized using the Integrative Genomics Viewer (IGV; [30]).

Differential expression (DE) was analyzed using DESeq2 1.16.1 [31]. The adjusted p-value (Benjamini-Hochberg method) cutoff for DE was set at < 0.05, log2 fold change of expression (LFC) cutoff was set at > 1. We performed GO term and pathway overrepresentation analyses on relevant lists of genes using the PANTHER gene list analysis tool with Fisher’s exact test and FDR correction [32].

### Transmission electron microscopy

Mature sperm and testicular tissue were washed with PBS, fixed in 3% glutaraldehyde at 4°C overnight, then washed with 0.1 M cacodylate buffer followed by fixation with 2% osmium tetroxide at 4°C for 2h. After dehydration and processing samples were embedded in Epon C (70°C, 48 h). Ultra-thin sections were contrasted with uranyl acetate and lead citrate and then examined and imaged with a scanning electron microscope (Crossbeam 550 FIB SEM, Zeiss, Germany) equipped with a retractable STEM detector in the Microscopy Core Facility of the University of Bonn.

### Sperm nuclear morphology

Nuclear morphology was analyzed for 3 individuals per genotype. Approximately 1.5 x 10^6^ sperm were fixed in methanol-acetic acid (3:1), spread onto a slide and stained with 4′,6-diamidino-2-phenylindole (DAPI) nuclear stain (ROTIMount FluorCare DAPI (Carl Roth GmbH, Karlsruhe, Germany)). At least 200 stained sperm cells per individual were imaged at 100x magnification using a Leica DM5500 B fluorescent microscope. Nuclear morphology was analyzed using the stand-alone version of the Nuclear Morphology program by Skinner et al. [33]. The program allows for automated detection and morphological analysis of mouse sperm nuclei (among other species and cell types). It additionally provides options for clustering heterogenous populations by nuclear parameters and comparative analyses of nuclear morphology [33].

### Sperm basic nuclear protein extraction and analysis

Basic nuclear proteins were extracted described in Soler-Ventura et al. [34]. This protocol is intended for the extraction of basic nuclear proteins from mature sperm, however non-nuclear proteins with properties that are compatible with the extraction conditions will also be present in the sample. Briefly, approximately 10 x 10^6^ of swim-out sperm were washed in PBS and pelleted. The pellet was resuspended in buffer containing 1M Tris pH 8, 0.5M MgCl and 5ul Triton X-100. Subsequently the pellet was treated with 1mM PMSF in water inducing cell lysis. Treatment with EDTA, DTT and GuHCl induced DNA denaturation. Incubation at 37°C for 30min in presence of 0.8% vinylpyridine is necessary for mouse protamine separation on the subsequent protein gel. DNA is then precipitated by addition of EtOH and separated from the sample by centrifugation. Basic sperm nuclear proteins are then extracted and dissolved in 0.5M HCl, followed by protein precipitation with TCA, acetone washes and drying. The precipitated proteins are resuspended in sample buffer containing 5.5 M urea, 20% β-mercapto-ethanol and 5% acetic acid. For direct comparison of protamine content between genotypes sperm of 4 to 5 individuals were pooled, counted and extraction performed as described above. The extracted proteins were then resuspended in sufficient sample buffer to achieve a concentration equivalent to 700K sperm per μl of extraction.

The samples were then run on a pre-electrophorized acid-urea polyacrylamide gel (AU-PAGE) (2.5 M urea, 0.9 M acetic acid, and 15% acrylamide/0.1% N,N′-Methylene bis-acrylamide, TEMED and APS). The extracted basic nuclear proteins migrate towards the negative pole at a 150V for 1h, 50min. For the evaluation of the protamine content the same amount of sample (equivalent to 3M pooled sperm) was loaded for each genotype to ensure comparability. The gels were stained with Coomassie Brilliant Blue (Sigma Aldrich, Taufkirchen, Germany) using standard procedures. The two main protamine bands can be observed in the bottom of the gel with mature-PRM2 corresponding to the upper and PRM1 the lower band [34,35]. PRM2 precursor bands can be observed in the lower part of the gel above the mature-PRM2 band, if present [22,36]. In the upper half of the gel, bands corresponding to other basic nuclear proteins, including histones can be found (see [34]). The densities of Coomassie stained bands were analyzed using ImageJ (1.52k, [37]).

### Custom cP2 antibody production

A cP2-specific custom antibody (Davids Biotechnologie GmbH; Regensburg, Germany) was developed to assess the presence of the PRM2 precursor and the timing of PRM2 processing. The antibody was raised in rabbit against the cP2 peptide sequence (MRSPSEGPHQGPGQDHEREEQGQGQGLSPERVEDYGRTH) and affinity purified.

### Western blot analysis

For immunoblotting of AU-PAGE, an equivalent of 1.5M sperm of pooled basic protein extraction was used. The proteins were transferred towards the negative pole onto a PVDF membrane (pore size 0.45μm; Roth, Karlsruhe Germany), blocked and incubated with the primary antibody overnight (antibody dilutions shown in S3 Table). For SDS PAGE, proteins were transferred towards the positive pole onto a PVDF membrane (pore size 0.45μm; Roth, Karlsruhe Germany). The membranes were then washed and incubated with an HRP-labelled secondary antibody followed by chemiluminescent detection using Westar NOVA 2.0 chemiluminescent substrate (Cyanagen, Bologna, Italy) or SuperSignal West Femto Maximum Sensitivity Substrate (Thermo Fisher Scientific, Waltham, USA). Since Merges et al. [38] was able to show that (outer dense fiber protein 2) ODF2, which is a major component of the sperm tail, is compatible with AU-PAGE and represents one of the prominent bands in the upper part of the Coomassie AU gel we used the protein as a loading control.

### Immunohistochemistry

PFA fixed testis and epididymis sections as well as EtOH fixed tubule preparations were used for immunofluorescent staining. Sections were deparaffinized in xylol and rehydrated. Sections and tubule preparations were washed in PBS and blocked for 30 min with normal horse serum (Vectorlabs, Burlingame, USA) at room temperature, followed by heat-activated antigen retrieval at pH6 and primary antibody incubation over night at 4°C. Antibodies and dilutions are shown in S3 Table. Slides were then double-stained with fluorescent secondary antibodies using the VectaFluor Duet Immunofluorescence Double Labeling Kit, DyLight 594 Anti-Rabbit (red), DyLight 488 Anti-Mouse (green) (Vectorlabs, Burlingame, USA), DAPI counterstained and coverslipped with ProLong Gold antifade reagent with DAPI (Thermo Fisher Scientific, Waltham, USA).

### Mass spectrometry and differential protein abundance analysis

Basic nuclear protein extractions were done for 3 individuals per genotype using approximately 10^6^ sperm. Extracted proteins were dissolved in sample buffer (5.5 M urea, 20% β-mercapto-ethanol and 5% acetic acid).

Peptide preparation: Protein solutions (5.5 M urea, 20% 2-mercapto-ethanol, 5% acetic acid) were dried in a vacuum concentrator and subjected to in solution preparation of peptides. Proteins were dissolved in 50 mM acrylamide solution (Tris-HCl, pH = 8) and alkylated for 30 min at RT. 1 μg of Trypsin were added for o/n proteolysis at 37°C. Dried peptides were dissolved in 10 μL 0.1% trifluoro acetic acid (TFA) and desalted with ZipTips (Waters GmbH, Eschborn, Germany) according to standard solid-phase extraction procedures. Equilibration and binding was done in presence of 0.1% TFA, washing with 0.1% formic acid (FA). Eluates (50% acetonitrile, 0.1% FA) were dried and stored at -20°C.

LC-MS measurements: Peptide separation was performed on a Dionex Ultimate 3000 RSLC nano HPLC system (Dionex GmbH, Idstein, Germany). The autosampler was operated in μl-pickup mode. Peptides were dissolved in 10 μl 0.1% FA (solvent A). 2 μL were injected onto a C18 analytical column (300 mm length, 75 μm inner diameter, ReproSil-Pur 120 C18-AQ, 1.9 μm). Peptides were separated during a linear gradient from 2% to 35% solvent B (90% acetonitrile, 0.1% FA) within 90 min at 300 nl/min. The nanoHPLC was coupled online to an Orbitrap Fusion Lumos mass spectrometer (Thermo Fisher Scientific, Bremen, Germany). Peptide ions between 300 and 1600 m/z were scanned in the Orbitrap detector every 3 seconds with R = 120,000 (maximum fill time 50 ms, AGC target 400,000). Polysiloxane (445.12002 Da) was used for internal calibration (typical mass error ≤1.5 ppm). In a top-speed method, peptides were subjected to higher energy collision induced dissociation (HCD: 1.0 Da isolation, threshold intensity 25,000, normalized energy 27%) and fragments analyzed in the Orbitrap with target 50,000 and maximum injection time 22 ms, R = 15,000. Fragmented peptide ions were excluded from repeat analysis for 20 s.

Data analysis: Raw data processing and was performed with Proteome Discoverer software 2.5.0.400 (Thermo Fisher Scientific). Peptide identification was done with an in-house Mascot server version 2.6.1 (Matrix Science Ltd, London, UK). MS data were searched against *Mus musculus* sequences from SwissProt (2021/03, including isoforms), and contaminants (cRAP, [39]). Precursor Ion m/z tolerance was 10 ppm, fragment ion tolerance 20 ppm. Tryptic peptides with up to two missed cleavages were searched. Propionamide on cysteines was set as static modification. Oxidation was allowed as dynamic modification of methionine, acetylation as modification of protein N-termini. Mascot results were evaluated by the percolator algorithm [40] version 3.05 as implemented in Proteome Discoverer. Spectra with identifications below 1% q-value were sent to a second round of database search with semitryptic enzyme specificity (one missed cleavage allowed). Protein N-terminal acetylation, methionine oxidation, carbamylation on lysine and N-termini were allowed as dynamic modifications. Actual FDR values were typically ≤0.5% (peptide spectrum matches), ≤1.0% (peptides), <1% (proteins). Proteins were accepted if at least two peptides with q-value <1% were identified. Summed abundances (areas of precursor extracted ion chromatograms of unique peptides) were used for relative quantification.

Differential abundance (DA) analysis: DA analysis was performed using the Bioconductor package proDA [41] using peptide spectrum matches (PSM) level data extracted from Protein Discoverer. Only proteins detected in all genotypes and all replicates with more than two peptides were included in the analysis. The data were log2 transformed and median normalized prior to DA analysis to ensure comparability. The proDA package is based on linear models and utilized Bayesian priors to increase power for differential abundance detection [41]. Proteins with a log2 fold change (LFC) of >1 and false discovery rate adjusted p-value (FDR) <0.05 were considered differentially abundant compared to the WT. Plots were generated using the R-package ggplot2 [42].

### Generation of expression plasmids and transfection

*Prm2*, cP2, Prm2^Δc^, *Tnp1* and *H2al2* cDNA (GenBank: NM_008933.2) was amplified from C57Bl6 mouse testis cDNA using overhang primers introducing suitable restriction enzyme motifs (S1 Table). *Prm2*, *cP2* and *Prm2*^*Δc*^ were cloned N-terminally in-frame with eGFP into the pEGFP-N3 vector (6080–1) (pPrm2-eGFP-N3; pCP2-eGFP-N3; pPrm2^*Δc*^-eGFP). Additionally, we generated a plasmid in which the cP2 sequence was tagged 3’ with a nuclear location signal (SV40-NLS: PKKKRKV) followed by eGFP (pCP2-NLS-eGFP-N3). cP2-NLS was also cloned N-terminally in frame with mCherry into the pMCherry-N1 vector (Clontech PT3974-5) (pCP2-NLS-mCherry-N1). *Tnp1* and *H2al2* were cloned N-terminally to mCherry into the pMCherry-N1 vector, however the stop codons were included to allow for expression of the sequences without tag (pTnp1-STOP-mCherry-N1, pH2al2-STOP-mCHerry-N1). Correct sequence and insertion were verified by sequencing. Schematic visualizations of the plasmids are shown in S2 Fig.

Human Embryonic Kidney 293 (HEK293) cells were cultured in standard medium (DMEM, 10% FBS). HEK293 cells were transfected at 80% confluence with 3μg of expression plasmid using FuGENE HD Transfection Reagent (Promega, Madison, USA), according to the manufacturer’s instructions. At 12 hours post-transfection the medium was changed. Cells were either harvested and proteins extracted or images were taken after 48 hours using a Leica DMIRB inverted microscope (Leica Microsystems, Wetzlar, Germany).

### GFP pull-down assay

48 hours post-transfection cells were harvested and proteins extracted using MPER mammalian protein extraction reagent (Thermo Fisher Scientific, Waltham, USA) followed by immunoprecipitation (IP) using GFP nanobody-coupled magnetic beads (GFP-Trap Magnetic Particles Kit, Chromotek GmbH, Munich, Germany) according to the manufacturer’s recommendations. After IP, the bound proteins were eluted in Laemmli buffer and analysed using SDS-PAGE followed by western blot (see above).

## Results

### Generation of gene-edited mouse lines

To delete cP2 in the reading frame of the *Prm2* gene, we used CRISPR/Cas9 with templates catalyzing homology directed repair (HDR). In order to induce the deletion, we used two gRNAs targeting the 5’ and 3’ ends of the cP2 domain (Figs 1A and S1A). A single stranded DNA template encoding the 5’ and 3’ areas flanking the cP2 coding region enabling deletion of the cP2 coding region and an in-frame repair, generating an allele, where only mature PRM2 is expressed from the endogenous promoter, was added to the gene-editing reaction (S1B Fig). The sequence of the generated allele, named Prm2^Δc^, is shown in S1C Fig and was registered with the mouse genomics database (MGI:6718282). Animals were generated, sequence validated and backcrossed to C57Bl/6J for at least 3 generations before analysis to ensure segregation of any possible distant off-target edits. Additionally, we evaluated the gene expression levels and mapping of RNAseq reads to the *Prm2* gene cluster (*Prm1*, *Prm2* and *Tnp2*) to exclude the possibility of off-target edits in the cluster or effects on gene expression regulation (S3 Fig).

### Prm2^+/Δc^ as well as Prm2^-/Δc^ male mice are infertile

First, we subjected the Prm2^+/Δc^ mice to a fertility test. Five Prm2^+/Δc^ male mice were mated to ten WT females and the pregnancy/litters were recorded for five confirmed vaginal plugs per male (successful matings). We found that Prm2^+/Δc^ male mice were infertile, with no observed pregnancies in at least five confirmed matings each. This is in contrast to the deletion of the entire PRM2 gene, where Prm2^+/-^ male mice remained fertile [6] (Fig 1B). We hypothesized that an aberrant interaction between the newly generated mP2 and the PRM2 precursor expressed from the wildtype allele might lead to interference and be causative for infertility in these males. In order to test this, we bred Prm2^+/Δc^ females with Prm2^+/-^ males published by us (Prm2^Δ97bp^; Schneider et al. 2016 [6], MGI:5760133) to generate Prm2^-/Δc^ mice. This results in male mice, in which only Prm2^Δc^ is present and expressed. However, Prm2^-/Δc^ males did not produce any litters in at least five confirmed matings each and can be considered infertile (Fig 1B). This strongly suggests, that the cP2 domain is essential for murine spermiogenesis. If cP2 was non-essential these Prm2^-/Δc^ mice should be fertile, similar to Prm2^+/-^ males.

### mP2 is expressed in Prm2^-/Δc^ mice and transcriptional silencing does not seem to be disrupted

Since the Prm2^-/Δc^ mice allow for detection and validation of mP2 transcripts from the Prm2^Δc^ allele we, performed RNAseq on testis samples and analyzed expression of *Prm2*. By mapping the RNAseq reads to the *Prm2* genomic location, we were able to verify that the mP2 transcript was indeed expressed from the gene edited Prm2^Δc^ allele (S4 Fig). The expression levels of the *Prm2* transcripts were comparable in all genotypes (WT, Prm2^+/Δc^ and Prm2^-/Δc^), thus the alleles do not display a gene dosage effect (Fig 1C).

In our previous study we found that in the Prm2^-/-^ testis, a much higher number of genes was differentially higher than lower expressed (81:13) indicating incomplete protamine-mediated transcriptional silencing [43]. Here, compared to WT, we found 36 genes differentially higher expressed and 42 genes differentially lower expressed for Prm2^+/Δc^ males and 26 genes differentially higher and 43 differentially lower for Prm2^-/Δc^ (Fig 1D). This indicates that transcriptional silencing is not notably disrupted in males harboring the Prm2^Δc^ allele. No GO-term or pathway enrichment was found for analyzed gene sets. Lists of differentially expressed genes and statistics can be found in S1 Data.

### mP2 is detected in Prm2^+/Δc^ and Prm2^-/Δc^ spermatid nuclei, but is also found in cytoplasm and residual bodies

**Fig 2:**
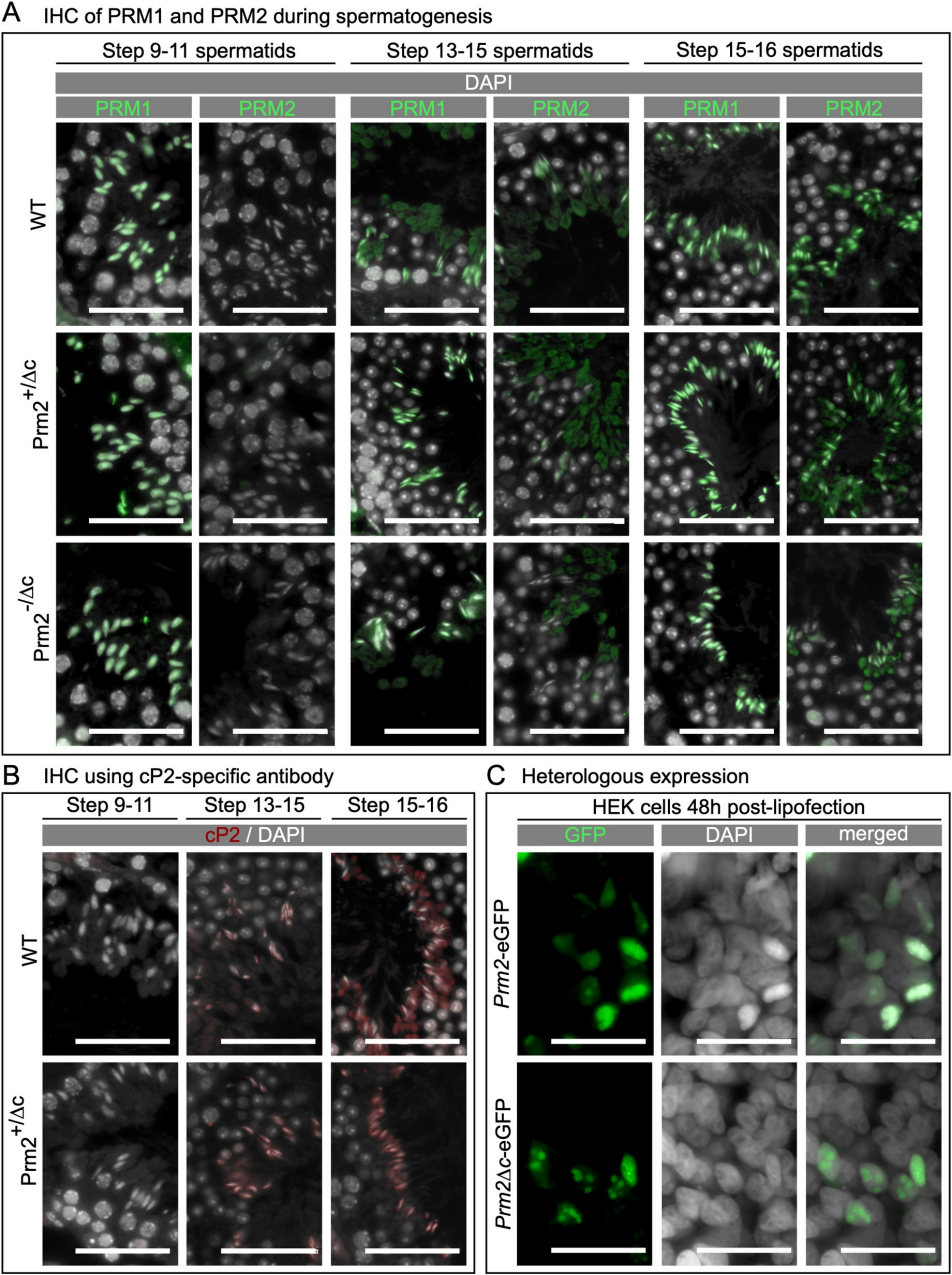
Localization and translation timing of PRM1, PRM2, unprocessed PRM2 and DNA condensing ability of Prm2^Δc^. (A) Immunohistochemical fluorescent staining of PRM2 (WT, Prm2^+/Δc^) or mP2 (Prm2^+/Δc^, Prm2^-/Δc^) (green) and PRM1 (all genotypes) (green) in testis sections, counterstaining with DAPI (pseudo-colored grey). Scale bar = 50μm. (B) Immunohistochemical fluorescent staining of unprocessed PRM2 (red) using a cP2-specific antibody in testis sections, counterstained with DAPI (pseudo-colored grey). Scale bar = 50μm. C) Fluorescent images of human embryonic kidney 293 (HEK) cells 48 hours post-transfection with plasmids encoding eGFP tagged PRM2 (*Prm2*-eGFP) or Prm2^Δc^ (Prm2^Δc^-eGFP) (green), counterstained with Hoechst (pseudo-colored grey). Scale bar = 50μm.

After confirming, that the gene-edited mP2-domain is expressed from the Prm2^Δc^ allele, we next addressed the question, whether mP2 can be detected in spermatids and is able to condense DNA. Since translational timing of protamines is tightly regulated to avoid premature chromatin condensation we also evaluated if PRM1 and 2 translational timing might be altered. We therefore first performed IHC on PFA fixed testis sections. The epitope of the PRM2 antibody (Hup2B, Briarpatch Bio, Livermore, USA) is located in the first half of the mP2 domain and is able to detect both, the PRM2 protein generated from the wildtype and the gene-edited Prm2^Δc^ allele. As shown in Fig 2A, PRM2 is detected starting from step 13–15 spermatids, while PRM1 can be detected as early as steps 9–11. This is consistent with previous findings and therefore suggests that PRM1 and 2 translational timing is not altered [44]. PRM1 and 2 are detected in condensed spermatid nuclei of Prm2^+/Δc^ and Prm2^-/Δc^ males, similar to the wildtype. However, strikingly, we additionally detected a PRM2 signal in the cytoplasm of spermatids and residual bodies that is strongest in Prm2^-/Δc^ males and not found in wildtype (Fig 2A). This indicates that Prm2^Δc^ cannot be correctly incorporated into sperm chromatin.

In order to analyze the timing of PRM2 processing in WT and Prm2^+/Δc^ and the absence of cP2 in Prm2^-/Δc^ mice we used a custom cP2-specific antibody. Consistent with IHC using the commercial PRM2 antibody (mP2 specific epitope), in WT the PRM2 precursor can be detected starting from step 13–15 spermatids (Fig 2B). However, the PRM2 precursor seems to be present almost exclusively in residual cytoplasm in condensed spermatids. This suggests that unprocessed PRM2 is present throughout spermiogenesis but remains in the residual bodies after spermiation, while only mature PRM2 is present in the nucleus. Given that the precursor is detected in the nucleus in earlier stages, it seems that unprocessed PRM2 is evicted from the nucleus during the last steps of spermatid development (Fig 2B). In Prm2^+/Δc^ males the timing of cP2 detection is similar to the WT, however, the PRM2 precursor does not seem to be correctly evicted from condensed spermatids and remains in the nucleus instead (Fig 2B).

### Prm2 and Prm2^Δc^ are able to condense DNA in somatic cells in culture

To determine if Prm2^Δc^ is able to condense DNA, we expressed PRM2 and the mP2 sequence of the Prm2^Δc^ allele tagged with eGFP in HEK293 cells. The ability of PRM1 to bind to DNA and condense nuclei when expressed *in vitro* in somatic cells had been demonstrated before by Iuso et al. [45] in sheep fibroblasts. Moritz et al. [46] showed that mP2 is able to bind and condense DNA strands using electrophoretic mobility shift and DNA curtain assays. The DNA condensation ability of mP2 has however, not been tested in somatic cell culture. Our experiments revealed that PRM2-eGFP and PRM2^Δc^-eGFP locate to the HEK293 cell nuclei and are present after 48h as large speckles in the nucleus, while a few nuclei seem to be fully condensed (Figs 2C and S5A). A western blot against GFP of HEK cells transfected with eGFP, Prm2-eGFP and Prm2^Δc^-eGFP seems to indicate that PRM2-eGFP might be cleaved in HEK cells. However, further experiments will be needed to confirm this result (S5B Fig). We therefore concluded that mP2 is able to condense the nucleus of somatic cells *in vitro* and should therefore be able to contribute to spermatid chromatin condensation *in vivo*.

To evaluate the behavior of the cP2 domain *in vitro* we transfected HEK293 cells with eGFP tagged cP2. cP2-eGFP is detected in the whole cell (S5C Fig), we therefore included a nuclear location signal (NLS) which leads to almost complete nuclear localization of cP2. cP2 does not seem to bind to DNA or initiate chromatin condensation (S5C Fig). When cP2-mCherry is co-transfected with mP2-eGFP, they do not co-localize and cP2 does not seem to take part in mP2 initiated chromatin condensation (S5C Fig). We therefore conclude that, *in vitro*, cP2 seems to only perform its intended function when part of PRM2.

### Testis histology is inconspicuous, while mature sperm are inviable and immotile in Prm2^+/Δc^ and Prm2^-/Δc^ male mice

**Fig 3:**
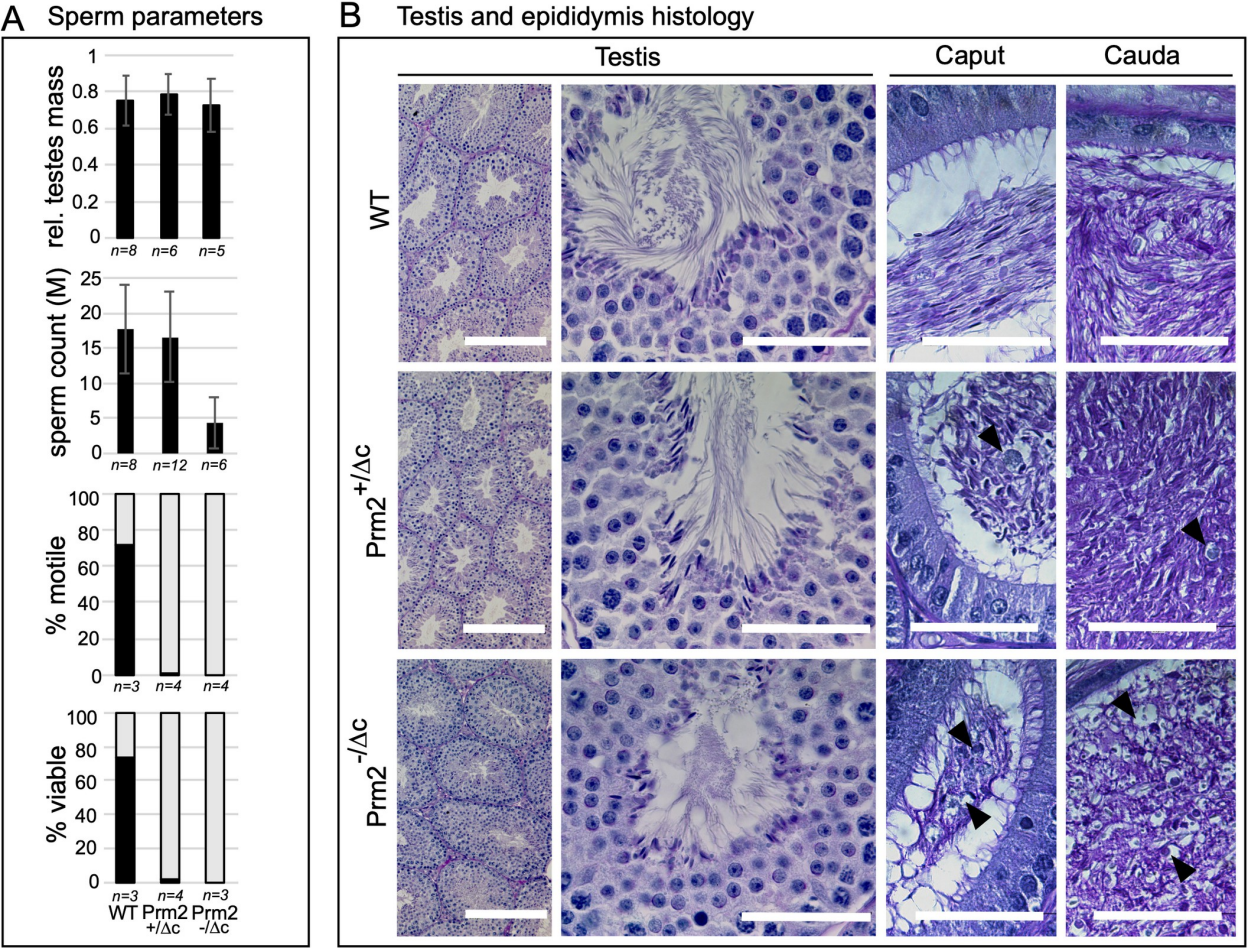
Sperm and testis parameters and histology. (A) Bar plots showing data for relative testes mass, mature sperm count, percentage of viable mature sperm (eosin-nigrosin assay) and percentage of motile mature sperm in Prm2^+/Δc^ and Prm2^-/Δc^ mice compared to wildtype. (B) PAS staining of testis and epididymal sections of Prm2^+/Δc^, Prm2^-/Δc^ and WT males. Scale bar = 50μm (200μm for left column).

Next, we analyzed testis mass and examined histological sections to determine the nature of the infertility. Relative testes mass did not differ from the wildtype (Fig 3A and S2 Table). Almost no viable and motile mature sperm were found in Prm2^+/Δc^ and Prm2^-/Δc^ males (Mean percent viable: Prm2^+/Δc^ = 1.7, SD = 1.31; Prm2^-/Δc^ = 0; Mean percent motile: Prm2^+/Δc^ = 0.2, SD = 0.45; Prm2^-/Δc^ = 0.25, SD = 0.5) (Figs 3A and S6 and S2 Table). Interestingly, sperm count is significantly reduced in Prm2^-/Δc^ but not in Prm2^+/Δc^ males compared to the wildtype (ANOVA: F(2) = 10.87, p<0.001; Post-hoc Tukey HSD: WT vs. Prm2^+/Δc^: p = 0.89, WT vs. Prm2^-/Δc^: p<0.001) (Fig 3A and S2 Table). To determine if the loss of cP2 affects spermiogenesis, testis and epididymis histology was evaluated by PAS staining of Bouin-fixed sections. Testis histology was inconspicuous and spermatogenesis seemed not to be affected in either genotype (Fig 3B). Epididymis histology however, shows larger round cells and vacuole-like structures, which are indicative of spermatid degradation. This was more pronounced in Prm2^-/Δc^ males compared to Prm2^+/Δc^ males (Fig 3B).

### Chromatin integrity is strongly affected starting in condensed spermatids in Prm2^+/Δc^ and Prm2^-/Δc^ male mice

**Fig 4:**
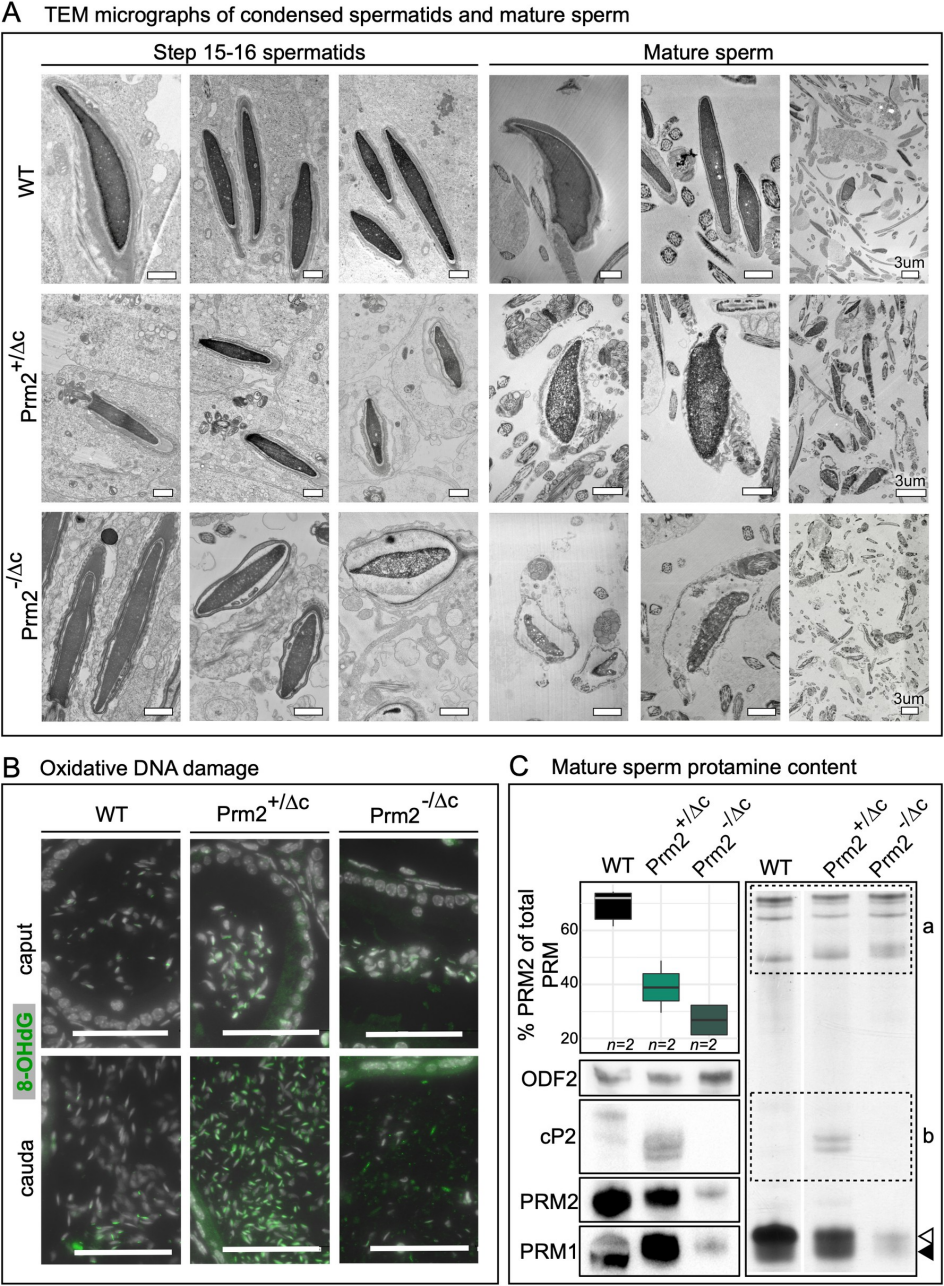
Chromatin integrity, oxidative damage and protamine content. (A) Electron micrographs of condensed spermatids and mature sperm of Prm2^+/Δc^, Prm2^-/Δc^ and WT. Scale bars = 1μm unless otherwise indicated. (B) Immunohistochemical fluorescent staining of 8-Oxo-2’-deoxyguanosine (8-OHdG) (green) in caput epididymis (upper row) and cauda epididymis (lower row) of Prm2^+/Δc^, Prm2^-/Δc^ and WT. Counterstained with DAPI (pseudo-colored grey). Scale bar = 50μm. (C) Boxplot showing the percentage of PRM2 (including PRM2 precursors) of total protamine by band density analysis of Coomassie stained acid urea gel electrophoresis (AU-PAGE) (S8C Fig). Asterisk indicates significant difference. To the right: AU-PAGE of WT, Prm2^+/Δc^ and Prm2^-/Δc^ mature sperm basic nuclear protein extractions, equal amounts of pooled sample loaded per genotype. a = non-protamine basic proteins, b = PRM2 precursors, open arrowhead indicates mature PRM2 band, solid arrowhead indicates PRM1 band. Below: Immunoblot of PRM1, PRM2, cP2 (indicating the presence of PRM2 precursor) and ODF2.

Since protamines condense sperm chromatin and were shown to influence sperm head morphology [20,47], mature sperm DNA integrity and nuclear morphology could be affected even though mP2 is able to condense DNA. We therefore extracted DNA from mature sperm and subjected it to agarose gel electrophoresis. DNA from sperm of Prm2^+/Δc^ males is completely fragmented (S7A Fig). TEM micrographs show nuclear envelope detachment and membrane and DNA degradation starting in condensed spermatids in the testis, which ultimately leads to complete membrane and DNA degradation in 100% of mature sperm in both genotypes (a minimum of 200 mature sperm observed in 2 individuals per genotype). The degradation appears more severe in Prm2^-/Δc^ than in Prm2^+/Δc^ males (Fig 4A). Schneider et al. [43] was able to show that during epididymal transit, Prm2^-/-^ deficient sperm underwent ROS mediated destruction, leading to DNA and membrane degradation and immotility. We therefore stained sections of epididymides against 8-Oxo-2’-deoxyguanosine (8-OHdG), which indicates oxidative DNA damage. In the caput epididymis we detected only a slight increase of the 8-OHdG signal in Prm2^+/Δc^ and Prm2^-/Δc^ males compared to the wildtype. However, in the Prm2^+/Δc^ cauda epididymis a strong increase in 8-OHdG was visible compared to the wildtype. The 8-OHdG signal in the Prm2^-/Δc^ cauda was less intense, which is likely due to the more severe degradation and reduced sperm count (Fig 4B).

Nuclear morphology analysis revealed aberrant nuclear morphology of mature sperm in Prm2^+/Δc^ and Prm2^-/Δc^ males. Sperm from both genotypes show a significantly reduced nuclear size compared to the WT (S7B Fig and S2 Table). Prm2^+/Δc^ males show two clusters of nuclear shape, a slimmer nucleus with decreased hook curvature and a smaller hookless nucleus. This phenotype is even more severe in Prm2^-/Δc^ males, which completely lack the cP2 domain (S7B Fig and S2 Table). Of note, nuclear morphology of Prm2^-/Δc^ and Prm2^+/Δc^ deficient sperm also differs from Prm2^+/-^ and Prm2^-/-^ males.

### The protamine ratio is flipped in Prm2^+/Δc^ and Prm2^-/Δc^ males

Since the ratio between PRM1 and PRM2 is constant in mature sperm (in mice ~60% PRM2), and alterations of this ratio are associated with sperm defects and infertility [48–50], we next tested if male mice harboring the Prm2^Δc^ allele display alterations of the PRM1/PRM2 ratio. Consistent with finding in IHC, acid-urea gel electrophoresis (AU-PAGE) of mature sperm basic nuclear proteins showed several bands corresponding to PRM2 precursors in Prm2^+/Δc^ males (Fig 4C). This was also the case in Prm2^+/-^ males (S8A Fig).

Comparing the densities of the PRM1 band relative to mP2 and PRM2 precursor bands, we found the PRM ratio to be strongly altered, showing a lower percentage of PRM2 (including precursors) compared to the wildtype in Prm2^+/Δc^ and Prm2^-/Δc^ males (mean %PRM2: WT = 69,29; Prm2^+/Δc^ = 42,07; Prm2^-/Δc^ = 26.78) (ANOVA: F(2) = 18.98,p = 0.009; Post-hoc Tukey HSD: WT vs. Prm2^+/Δc^: p = 0.04, WT vs. Prm2^-/Δc^: p = 0.008). This does not seem to be the case in Prm2^+/-^ males, for which we find the percentage of PRM2 (including precursors) to be similar to the wildtype (%PRM2 = 66.57 (n = 1)) (Figs 4C and S8 and S2 Table).

By loading the same amount of pooled sperm basic protein extraction for each genotype we were able to reliably compare total protamine content by Coomassie staining and western blot. This revealed that in addition to the alteration of the protamine ratio, the amount of both protamines is severely reduced in Prm2^-/Δc^ males (Figs 4C, S8 and S9). This is already evident in the caput epididymis (S8B Fig). Of note, the PRM2 precursor is not detectable in WT mature sperm Coomassie stained AU gels since only mature PRM2 remains bound to completely condensed sperm chromatin. However, a very small amount of PRM2 precursor can be still observed in WT mature sperm in western blot (Fig 4C).

These data, together with the mP2 signal detected in condensing spermatid cytoplasm (Fig 2A), strongly suggest, that loss of the cP2 domain leads to a severe reduction of PRM2 and a moderate loss of PRM1 associated with DNA.

### Histone-to-protamine transition is incomplete in Prm2^+/Δc^ and Prm2^-/Δc^ males

**Fig 5:**
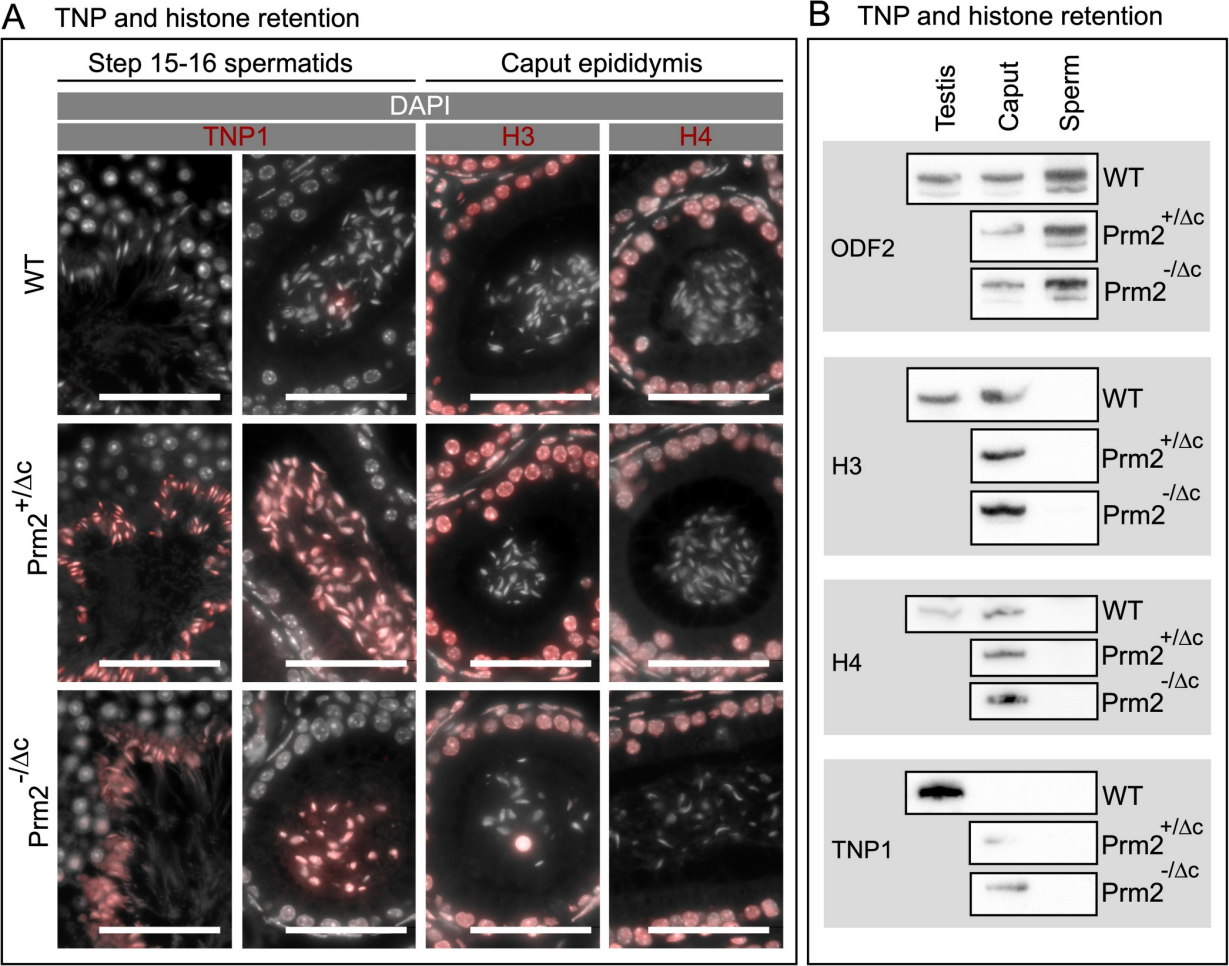
Histone H3, histone H4 and transition protein 1 IHC staining and western blot detection. (A) Two left columns: Immunohistochemical fluorescent staining of TNP1 (red) in WT, Prm2^+/Δc^ and Prm2^-/Δc^ step 15–16 spermatids and caput epididymis sections, counterstained with DAPI (pseudo-colored grey). Scale bar = 50μm. Two right columns: Immunohistochemical fluorescent staining of Histone H3 (H3) or Histone H4 (H4) (red) in WT, Prm2^+/Δc^ and Prm2^-/Δc^ caput epididymis sections counterstained with DAPI (pseudo-colored grey). Scale bar = 50μm. (B) Immunoblots against TNP1 and H3 and H4 and ODF2 as a control in testis (WT), caput epididymis (caput) and mature sperm (sperm) basic nuclear protein extractions (WT, Prm2^+/Δc^ and Prm2^-/Δc^).

Since we found the relative level of PRM2 to be reduced, and PRM2 aberrantly located in the cytoplasm and residual bodies of condensing/condensed spermatids in the testis, we next evaluated the histone-to-protamine transition. To this end, we performed IHC staining and western blots of basic nuclear protein extractions to detect histone H3, histone H4 and transition protein 1 (TNP1) in testis and epididymis. We did not find any apparent increase in total histone H3 or histone H4 signal (Figs 5A and 5B, and S9), However, in contrast to WT, TNP1 was retained in both Prm2^+/Δc^ and Prm2^-/Δc^ males in step 15–16 spermatids and caput epididymal sperm. In cauda epididymal sperm however, we did not find any visible signal of TNP1 (Figs 5A, 5B and S9). This demonstrates that loss of cP2 leads to transition protein retention.

**Fig 6:**
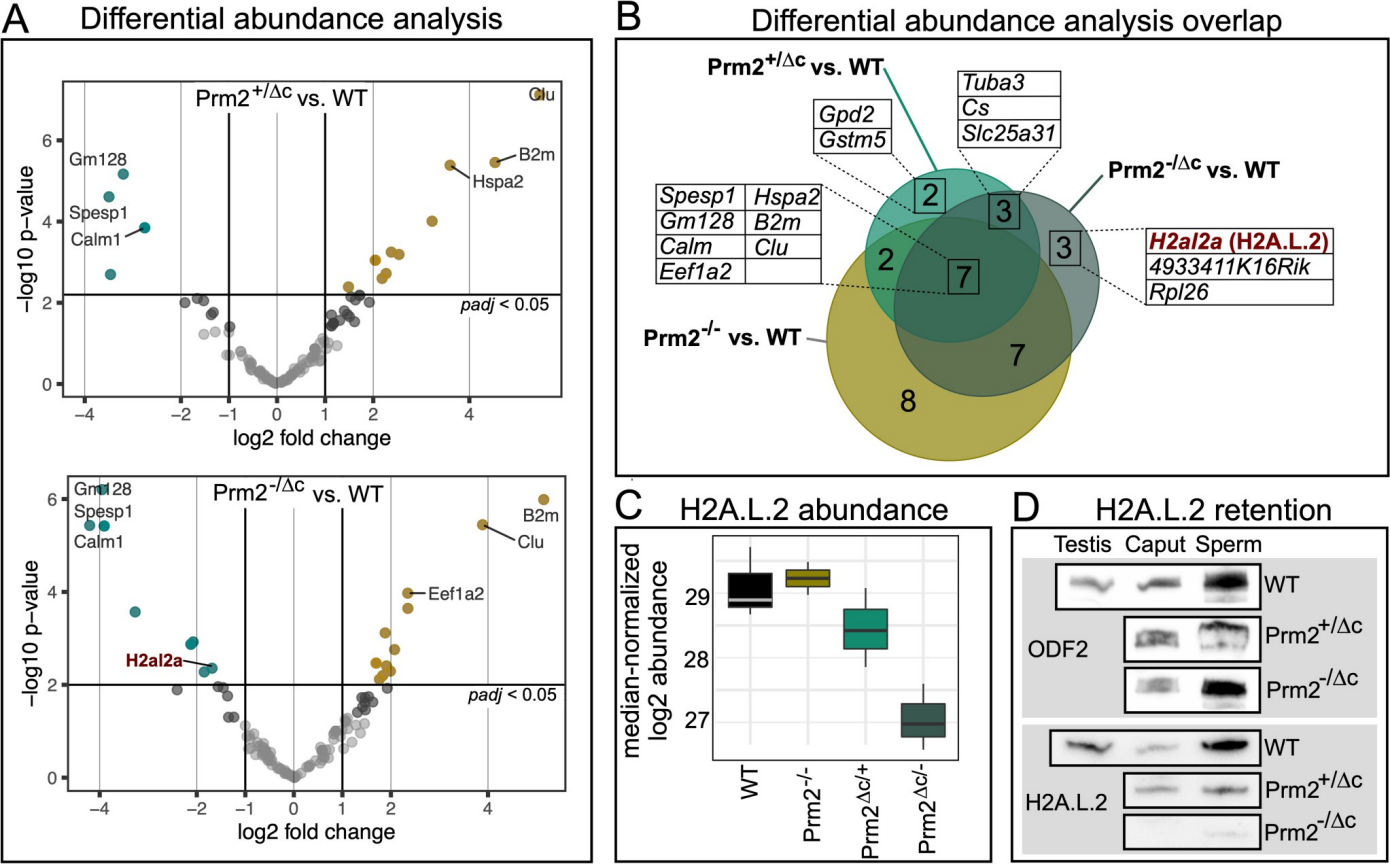
Differential abundance of mature sperm basic nuclear proteins. (A) volcano plots showing differential abundance (DA) of basic nuclear proteins in Prm2^+/Δc^ compared to WT (upper plot) and Prm2^-/Δc^ compared to WT (lower plot). Significantly DA proteins are indicated in color (teal = lower abundant, yellow = higher abundant). Top DA proteins and proteins of interest are labeled with their corresponding gene symbol. (B) Venn diagram showing the overlap between DA proteins found in the three different comparisons (WT vs. Prm2^+/Δc^, WT vs. Prm2^-/Δc^ and WT vs. Prm2^-/-^). Proteins present in overlaps of interest are listed with their corresponding gene symbol. H2A.L.2 is marked in red. (C) Boxplot of median normalized log2 abundance of H2A.L.2 in WT, Prm2^-/-^, Prm2^+/Δc^ and Prm2^-/Δc^. (D) Immunoblots against H2A.L.2 and ODF2 as a control in testis (WT), caput epididymis (caput) and mature sperm (sperm) basic nuclear protein extractions (WT, Prm2^+/Δc^ and Prm2^-/Δc^).

In order to further investigate histone retention and alteration in nuclear protein content we performed mass spectrometric analysis on mature sperm basic nuclear protein extracts and analyzed differential abundance (DA) of the detected proteins. Compared to wildtype, we found 14 proteins to be DA in Prm2^+/Δc^ sperm, 20 in Prm2^-/Δc^ males and 24 for Prm2^-/-^ sperm. Seven proteins were DA in all three comparisons, several of those associated with stress response and/or apoptosis (HSPA2, B2M, CLU) (Fig 6A and 6B). Consistent with IHC results we did not find any histones that were significantly higher abundant in Prm2^+/Δc^ or Prm2^-/Δc^ males. We therefore conclude that histone retention is not increased in males harboring the Prm2^Δc^ allele. Interestingly in the Prm2^-/-^ samples we did find histones (H3f3, H3C, H4C) to be significantly higher abundant, indicating increased histone retention when PRM2 is completely lacking. Transition proteins and protamines were not detected due to technological limitations. Lists of DA proteins including statistics can be found in S2 Data.

Eight proteins were not DA in Prm2^-/-^ compared to WT, but in Prm2^+/Δc^ and/or Prm2^-/Δc^ males. Of these, RPL26 and GSTM5 are involved in DNA damage response and/or oxidative stress pathways, while TUBA3B and ANT4 (*Slc25a31*) are related to motility. Of note, citrate synthase (CS) is specifically higher abundant in Prm2^+/Δc^ and Prm2^-/Δc^ males. Most interestingly, we found the histone H2A variant H2A.L.2 to be significantly lower abundant in Prm2^-/Δc^ males, compared to the wildtype (Fig 6A–6D). H2A.L.2 is a spermatid/sperm-specific histone variant and a key player in histone-to-protamine transition. Together with TH2B it forms a nucleosome with an open chromatin structure allowing for loading of transition proteins followed by protamine recruitment and histone and transition protein eviction by protamines [8]. We confirmed the reduction in abundance of H2A.L.2 by western blot and additionally show that this reduction is already present in caput epididymis (Fig 6D).

### H2A.L.2, TNP1 and unprocessed PRM2 localize in heterochromatin of condensed spermatids in cP2 deficient males

**Fig 7:**
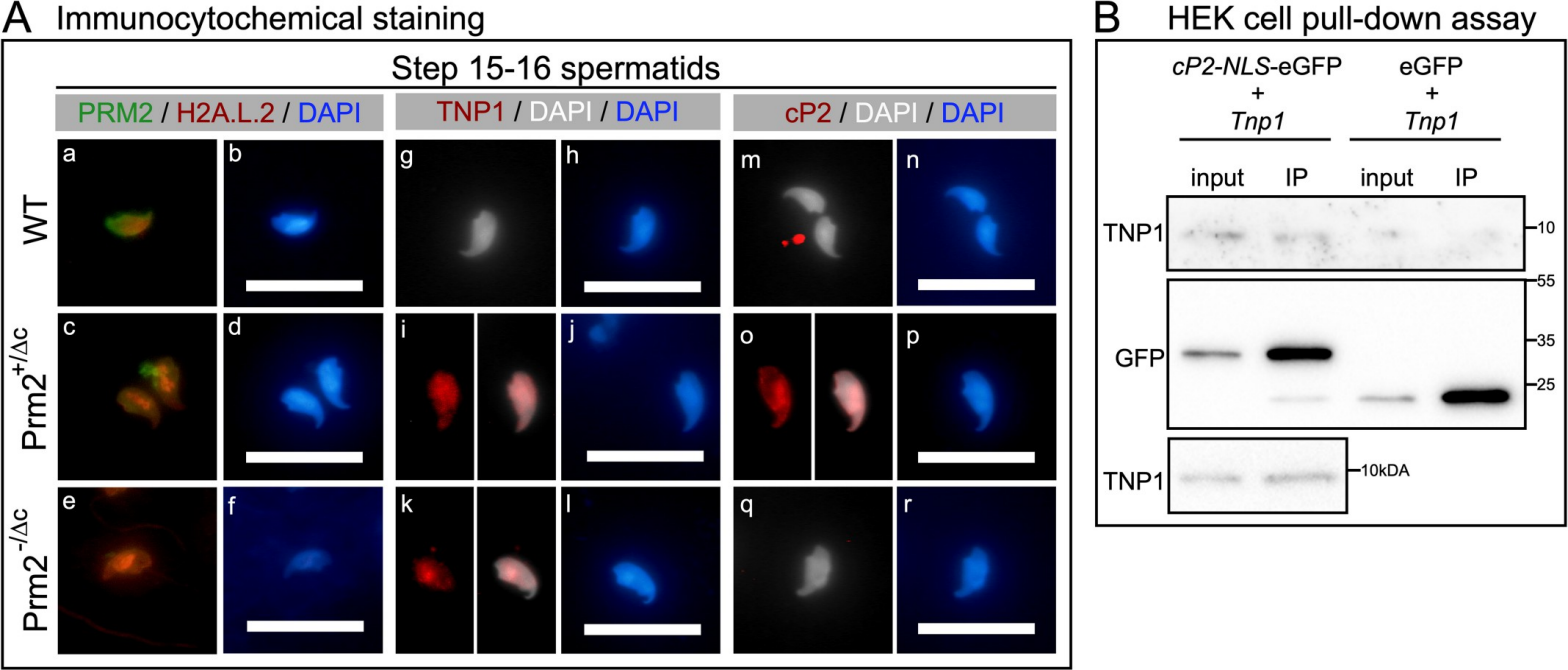
Immunocytochemical staining of H2A.L.2 and PRM2 as well as TNP1 and unprocessed PRM2. (A) (a-f) Immunocytochemical fluorescent staining of H2A.L.2 (red) and PRM2 (green) in WT, Prm2^+/Δc^ and Prm2^-/Δc^ step 15–16 spermatids from tubule preparations. Counterstained with DAPI (blue). (g-l) Immunocytochemical fluorescent staining of TNP1 (red) in WT, Prm2^+/Δc^ and Prm2^-/Δc^ step 15–16 spermatids from tubule preparations. Counterstained with DAPI (pseudo-colored grey or blue). (m-r) Immunocytochemical fluorescent staining of cP2 indicating the PRM2 precursor (red) in WT, Prm2^+/Δc^ and Prm2^-/Δc^ step 15–16 spermatids from tubule preparations. Counterstained with DAPI (pseudo-colored grey or blue). Scale bar = 20μm. (B) Immunoblots against GFP and TNP1 of GFP pull-down assay of HEK293 cells co-transfected with cP2-NLS-eGFP (pCP2-NLS-eGFP-N3) and untagged TNP1 (pTnp1-STOP-mCherry-N1) or eGFP (pEGFP-N3) and untagged TNP1. Immunoblot was repeated for cP2-NLS-eGFP and untagged TNP1 co-transfected samples (lower blot).

According to Hoghoughi et al. [51] H2A.L.2 is retained in mature sperm in pericentric heterochromatin. We therefore investigated the presence and co-localization of H2A.L.2, TNP1 and PRM2 in Prm2^-/Δc^ and Prm2^+/Δc^ males, by immunofluorescent staining of condensed step 15–16 spermatids from tubule preparations. In the WT, we found a moderately strong signal for H2A.L.2 in the pericentric region of the nucleus, with PRM2 signal in the whole nucleus (Fig 7A (a-b)). However, in Prm2^+/Δc^ and Prm2^-/Δc^ step 15–16 spermatids the H2A.L.2 signal is stronger compared to the WT and localized in DAPI-bright foci in the nucleus (Fig 7A (c-f)). DAPI-bright regions in nuclei usually correspond to heterochromatin. In mature sperm, however, the H2A.L.2 signal is almost completely lost in Prm2^+/Δc^ and Prm2^-/Δc^ males (S10A Fig), consistent with the lower abundance found in mass spectrometric analysis and western blot. Of note, the PRM2 signal is diffuse and distributed along the whole mature sperm cell, likely due to severe membrane damage and degradation (S10A Fig). TNP1 cannot be detected in WT condensed spermatids, in Prm2^-/Δc^ and Prm2^+/Δc^ however it is detected and seems to concentrate in DAPI bright regions similar to H2A.L.2 (Fig 7A (g-l)). Unprocessed PRM2 cannot be detected in the WT or Prm2^-/Δc^ males as expected. However, in Prm2^+/Δc^ a similar localization concentrating in DAPI-bright regions of the nucleus can be observed (Fig 7A (m-r)).

Finally, Co-transfection of cP2 tagged with an NLS signal and eGFP and untagged *Tnp1* in HEK293 followed by GFP pull-down indicated that the cP2 domain might interact with TNP1 *in vitro* (Figs 7B and S10B). This should be confirmed by further experiments.

## Discussion

The crucial role of protamines in the process of sperm chromatin reorganization is well known. However, why two protamines are needed in some species, while PRM1 seems to be sufficient in others is still unclear. The main difference between PRM1 and PRM2 is the highly conserved N-terminal cleaved PRM2 domain (cP2). Its function remains elusive to date. Using gene-editing we generated mice lacking the cleaved-*Prm2* (cP2) domain. We show that the cP2 domain is indispensable for PRM2 function and required for male mouse fertility. Mice heterozygous for the deletion of the domain display inviable and immotile sperm. Loss of cP2 leads to severe retention of transition proteins and reduced incorporation of PRM2 into nucleo-protamine. Instead, mature-PRM2 (mP2) is aberrantly found in spermatid cytoplasm and residual bodies. While overall histone retention is not increased, the histone variant H2A.L.2 is less abundant in mature sperm deficient in cP2.

Firstly, mP2 expressed from the Prm2^Δc^ allele was detected in the nucleus of condensed spermatids of cP2 deficient mice and was shown to be able to condense somatic cell DNA *in vitro* similar to PRM2, or PRM1 [45]. We therefore conclude that the mP2 domain produced by gene editing seems to maintain its main function during spermiogenesis in cP2 deficient mouse lines. Male Prm2^+/Δc^ mice are infertile, show complete fragmentation of mature sperm DNA, loss of viability and immotility of mature sperm. This is in contrast to PRM2^+/-^ males, which remained fertile [6]. In order to test, whether the infertility of Prm2^+/Δc^ mice is due to an aberrant interaction between mP2 and the wildtype PRM2 precursor, we bred the Prm2^Δc^ allele with the Prm2^Δ97bp^ mouse line generated and analyzed by Schneider et al. [6,43]. However, mice expressing only mP2 (Prm2^-/Δc^) were also infertile showing an even more extreme phenotype than Prm2^+/Δc^ males. Since Prm2^+/-^ male mice are reported to be fertile, these data clearly indicate, that loss of cP2 leads to male infertility in mice. We speculate, that this holds also true for all other species which harbor a functional PRM2 gene. Additionally, the infertility of Prm2^+/Δc^ strongly suggests a dominant effect of the deletion, possibly due to an aberrant interaction with another key protein such as transition proteins, PRM1 or H2A.L.2.

Since mature sperm chromatin is completely fragmented in cP2 deficient males and mature sperm are inviable and immotile similar to Prm2^-/-^ sperm, we suspected that sperm might undergo epididymal degradation mediated by oxidative stress. Schneider et al. [43] showed that loss of PRM2 seems to lead to reduced antioxidant capacity of sperm, initiating an oxidative stress-mediated destruction cascade during epididymal transit. Indeed, cP2 deficient mice showed a strong increase in oxidative DNA damage in the cauda epididymis. Prm2^-/Δc^ sperm seem to be degraded to an extent that many of the caudal sperm are completely disintegrated, leading to a significantly reduced sperm count. However, ultrastructural imaging of condensed spermatids indicates that the membrane and DNA degradation is initiated in the testis and then increases throughout epididymal transit. This initiation of degradation might be correlated to the aberrant eviction of mP2 from the nucleus during this stage of sperm development. Interestingly, and in contrast to Prm2^-/-^ mice, also in Prm1^-/-^ mice DNA damage can be detected in the testis already.

Given the timing of *Prm2* expression and processing, the primary effects of cP2 loss are likely to be found during the transition from histone-bound to protaminized DNA in the final stages of spermiogenesis. Barral et al. [8] describe this transition taking place by assembly of a histone (TH2B and H2A.L.2)—transition protein (TNP1 and TNP2) interface followed by protamine recruitment and processing. Protamines themselves subsequently replace histones. This process is disrupted in cP2 deficient mice.

Firstly, in cP2 deficient mice mP2 is detected in the cytoplasm and residual bodies in addition to the signal found in the nucleus, indicating that mP2 is not completely incorporated into the condensing chromatin. In consequence, the protamine ratio changes from 2:1 in wildtype to 1:2–1:5 in cP2 deficient sperm. Surprisingly, in addition to the altered ratio, in Prm2^-/Δc^ mice we found the amount of PRM1 to be also severely reduced in caput and cauda epididymal sperm. The loss of cP2 thus seems to affect the incorporation of both protamines. This indicates, that the cP2 domain facilitates incorporation of protamines and the assembly of nucleo-protamine.

Secondly, in Prm2^+/Δc^ mice unprocessed PRM2 can be detected in condensed spermatid nuclei and in caput and cauda epididymal sperm. While in WT condensed spermatids unprocessed PRM2 seems to accumulate in cytoplasm and residual bodies in Prm2^+/Δc^ mice it is detected in the nucleus and does not seem to be evicted into the cytoplasm. Incomplete PRM2 processing has been reported in several mouse models of genes involved in the histone-to-protamine transition, such as transition proteins, PRM1 and H2A.L.2 [8,21,23,38,46,52]. At least in this study, however, it seems that rather than failure of complete processing of PRM2 we see a failure of eviction of unprocessed PRM2 into the cytoplasm. If this is the case for the other mentioned mouse models remains to be determined.

Further, we found retention of TNP1 in condensed testicular spermatids and caput epididymal sperm, indicating that the eviction of TNP1 is hampered due to the loss of cP2 or that TNP1 is binding DNA in a competitive manner. Of note, a recent study investigating a single residue mutation in PRM1 showed increased histone retention, but no disturbances in transition protein retention in such mice [46]. In Prm1^-/-^ transition protein retention is also present, albeit weaker than seen here [38]. Transition proteins are believed to aid in chromatin condensation by stabilizing the DNA in a non-supercoiled state, cooperating with topoisomerases to relieve torsional stress and to be involved in DNA repair during chromatin condensation [53–55]. Loss of either transition protein results in incomplete PRM2 processing [21,52,56] and TNP2 was shown to interact with PRM2 [8]. This demonstrates that transition proteins are required for proper PRM2 processing during nucleo-protamine assembly. The fact that we find severe retention of TNP1 in condensed spermatids indicates, that the cP2 domain in turn, is required for the proper processing (i.e. eviction) of TNP1. Indeed, we were able to provide tentative evidence that cP2 might interact with TNP1. Our results show that cP2 tagged with eGFP seems to be able to pull down TNP1 in somatic cell culture. Further analyses will be needed to confirm these results and to determine if the mP2 domain is able to interact with TNP1 as well. Of note, Rezaei-Gazik et al. [57] have recently shown that unprocessed PRM2 co-localizes with TNP1 in elongating mouse spermatids. The fact that transition protein were also slightly retained in Prm1^-/-^ mice could be related to the strong presence of unprocessed PRM2 in these mice.

Surprisingly, unlike transition proteins, overall histone retention was not increased. This was confirmed by H3 and H4 immunostaining, western blot and mature sperm basic nuclear protein abundance analysis. Interestingly, we do find an increased retention of H3 and H4 variants in Prm2^-/-^ deficient sperm. This suggests, that the global amount of retained histones is unaffected in cP2 deficient mice, and hence not controlled by the protamine ratio or PRM2 processing.

However, the retention of transition proteins detected in cP2 deficient mice seems to go along with altered retention of H2A.L.2. During the first steps of chromatin condensation H2A.L.2, together with TH2B provides an open chromatin interface necessary for transition protein loading. Barral et al. [8] showed, that deletion of H2A.L.2 leads to infertility, aberrant transition protein loading and disturbed processing of PRM2. In mature sperm H2A.L.2 was shown to be retained in pericentric heterochromatin [51,58]. This retention seems to be lost in Prm2^-/Δc^ caput epididymal sperm, where H2A.L.2 was shown to be significantly lower abundant. However, a H2A.L.2 signal can still be observed in Prm2^-/Δc^ condensed spermatids in the testis, were it overlaps with atypical speckles of bright DAPI signal. A similar signal can be seen for TNP1 in Prm2^+/Δc^ and Prm2^-/Δc^ mice and for unprocessed PRM2 in Prm2^+/Δc^ mice. Thus, it seems that H2A.L.2-transition protein complexes (together with unprocessed PRM2, when present) are retained in aberrant clumps of heterochromatin in condensed spermatids of cP2 deficient mice, after which H2A.L.2 seems to be evicted or lost, in Prm2^-/Δc^, in which cP2 is completely absent.

Protamines have a strong electrostatic attraction to DNA due to arginine clusters [46]. This allows protamines to condense DNA even in the absence of spermatid specific histones and transition proteins, as shown in *in vitro* assays in somatic cells and in this study [45]. However, uncontrolled or unbalanced binding of protamines might lead to strong torsional stress and DNA damage. A controlled stepwise chromatin condensation therefore could be required to maintain chromatin integrity. Moritz et al. [46] recently showed that the PRM2 precursor has a lower DNA binding affinity than mP2, leading to faster DNA condensation by mP2. Barral et al. [8] suggested that transition proteins buffer protamine incorporation by allowing for ordered protamine loading and (or possibly through) PRM2 processing. By losing the cP2 domain this processing step is skipped. We therefore propose, that the aberrant interaction with transition proteins due to cP2 deficiency leads to random mP2 binding, leading to strong hypercondensation of open chromatin resulting in DNA strand breaks. Transition protein-loaded chromatin, however, cannot be freed without the interaction with the cP2 domain and does not allow for mP2 or PRM1 loading, leading to incomplete protamine incorporation and retention of H2A.L.2 –transition protein complexes in aberrantly located heterochromatin foci, that are lost when the cP2 domain is completely lacking, starting in the testis and being completed during epididymal transit.

In conclusion, our results show that the cleaved domain of PRM2 is essential for sperm function and fertility. Loss of the domain leads to incomplete protamination, a switch in the protamine ratio and transition protein retention in epididymal sperm. Starting in condensed spermatids, cP2 deficient sperm are degraded, leading to complete DNA fragmentation. cP2 seems to be necessary for correct interaction between the H2A.L.2 - transition protein complex and PRM2 and might directly interact with TNP1. We were able to provide a first glimpse into the function of cleaved PRM2 and PRM2 processing, that opens up multiple avenues for further investigation.

## Supporting information

S1 Fig Gene editing. A) Schematic representation of CRISPR/Cas9 mediated gene editing. Gene-edited coding sequence and predicted translation are shown. B) ssODN repair template used during CIRSPR/Cas9 gene editing to evoke HDR mediates double strand break repair, fixing the startcodon and reading frame. C) Gene-edited coding sequence, including intron and predicted translation compared to the WT sequence.
(TIFF)

S2 Fig Schematic view of representative sections of expression plasmids used in this study. (TIFF)

S3 Fig Barplot showing average DESeq2 normalized read counts of Tnp1, Tnp2 and Prm1 for WT, Prm2+/Δc and Prm2-/Δc. B) Representative cutout of mapping of RNAseq reads to the genes present in the Protamine cluster.
(TIFF)

S4 Fig Representative cutout of RNAseq read mapping to the *Prm2* gene locus. (TIFF)

S5 Fig Heterologous expression. A) Heterologous expression of plasmids encoding eGFP tagged PRM2 (Prm2-eGFP), mP2 (mP2-eGFP) or eGFP (pEGFP-N3) in human embryonic kidney 293 (HEK) cells 48 hours post-transfection. Scale bar = 50μm. B) Western blot against GFP (Anti-GFP antibody ab6556, 1:1000) of protein extraction from HEK cells transfected with GFP (pEGFP-N3), cP2 (cP2-NLS-eGFP), mP2 (Prm2Δc-eGFP) or Prm2-eGFP. C) Heterologous expression of plasmids encoding eGFP tagged cleaved-PRM2 (cP2-eGFP) or tagged cleaved-PRM2 including a nuclear location signal (NLS) (cP2-NLS-eGFP; cP2-NLS-mCherry). Lower panel shows heterologous co-expression of mP2 (mP2-eGFP) and cP2-NLS-mCherry in human embryonic kidney 293 (HEK) cells 48 hours post-transfection. White scale bar = 50μm; grey scale bar = 10μm.
(TIFF)

S6 Fig Representative images of eosin-nigrosin stained mature sperm. (TIFF)

S7 Fig Chromatin integrity, nuclear morphology and protamine content. A) Agarose gel of DNA extracted from WT, Prm2+/-, Prm2-/- and Prm2+/Δc mature sperm. B) Comparison of mature sperm nucleus consensus shapes of WT, Prm2+/-, Prm2-/-, Prm2+/Δc and Prm2-/Δc resulting from nuclear morphology analysis. Numbers inside the consensus shapes indicate the number of nuclei assessed and assigned to the respective consensus shape cluster. Upper row shows the consensus shape of the different gene edited lines overlaid with the wildtype consensus shape. cl. = cluster. Boxplot of nuclear head area calculated during Nuclear Morphology Analysis. Below: detection parameters used for nuclus detection in the Nuclear Morphology program [33]
(TIFF)

S8 Fig Acid-Urea PAGE coomassie stained pictures. A) Gels loaded with the same amount of pooled samples for evaluation protamine content. B) Acid-urea gel electrophoresis (AU-PAGE) of WT, Prm2+/Δc and Prm2-/Δc caput epididymis basic nuclear protein extractions. C) Lanes used for analysis of protamine ratio are indicated. Asterisk indicate lanes with samples belonging to different studies and not used in this study.
(TIFF)

S9 Fig Western blot membranes of acid-urea PAGE used in this study. (TIFF)

S10 Fig Immunofluorescent staining of mature sperm and western blots of SDS-PAGE from HEK cell pull-down assays. A) Immunohistochemical staining of H2A.L.2 and PRM2 in mature sperm extracted from cauda epididymis. Counterstained with DAPI (blue). Scale bar = 20μm. B) Immunoblots of transfected HEK cell protein extractions and HEK cell pull-down assays. IP = immuno-precipitation.
(TIFF)

S1 Table Oligo DNA sequences used in this study. (XLSX)

S2 Table Fertility, testes mass, sperm count, viability, motility, protamine 2 content and nuclear morphology numerical data. (XLSX)

S3 Table Antibodies and dilutions. (XLSX)

S1 Data Differential expression analysis data. (XLSX)

S2 Data Mass spectrometric and differential abundance data. (XLSX)
