# Supplementary figures and images for: Loss of the cleaved-protamine 2 domain leads to incomplete histone-to-protamine exchange and infertility in mice
Source: PLoS Genet. 2022 Jun 28;18(6):e1010272. doi: 10.1371/journal.pgen.1010272 (PMC9273070; doi:10.1371/journal.pgen.1010272)

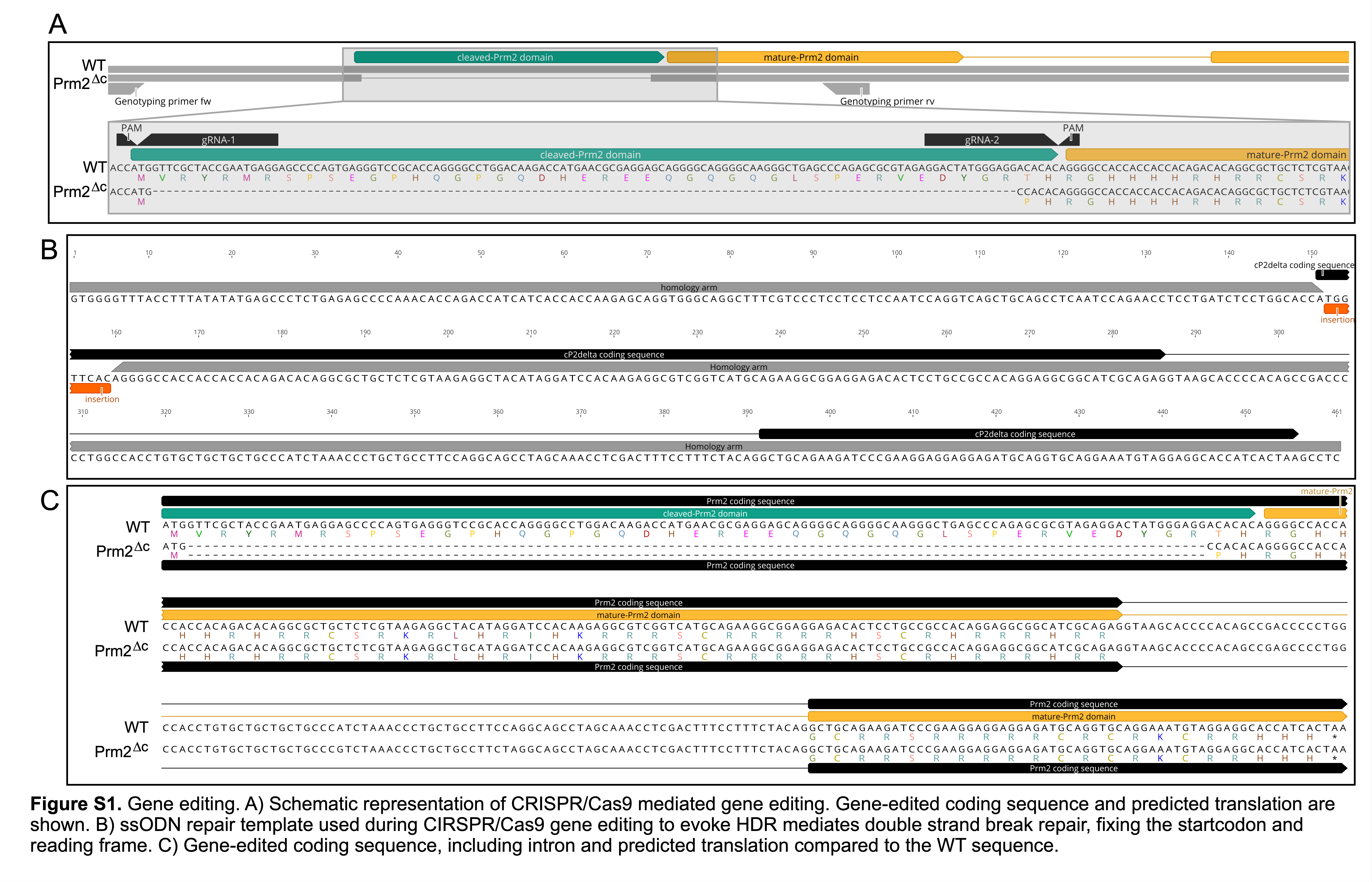

Supplement: S1 Fig — A) Schematic representation of CRISPR/Cas9 mediated gene editing. Gene-edited coding sequence and predicted translation are shown. B) ssODN repair template used during CIRSPR/Cas9 gene editing to evoke HDR mediates double strand break repair, fixing the startcodon and reading frame. C) Gene-edited coding sequence, including intron and predicted translation compared to the WT sequence. (TIFF) [file pgen.1010272.s001.tiff]

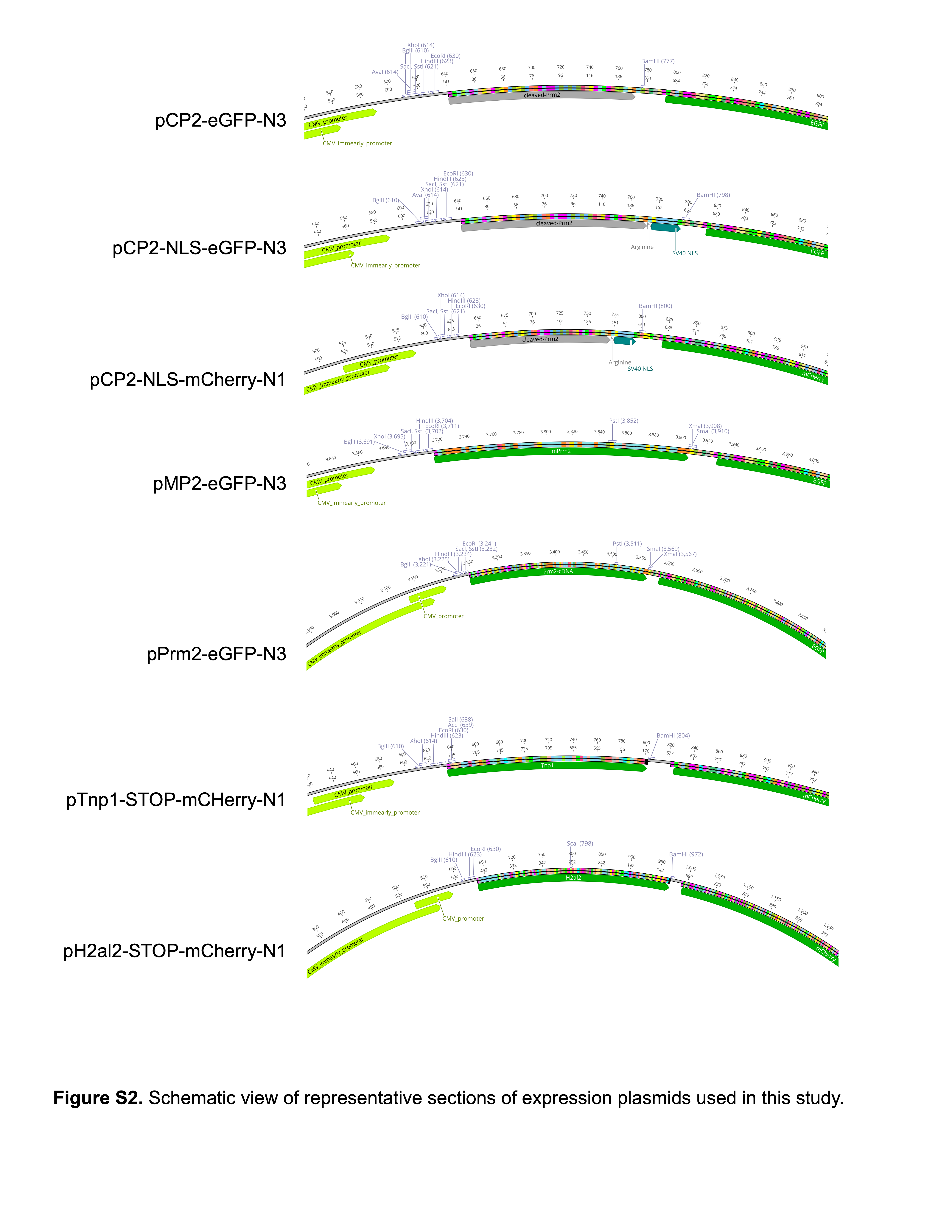

Supplement: S2 Fig — (TIFF) [file pgen.1010272.s002.tiff]

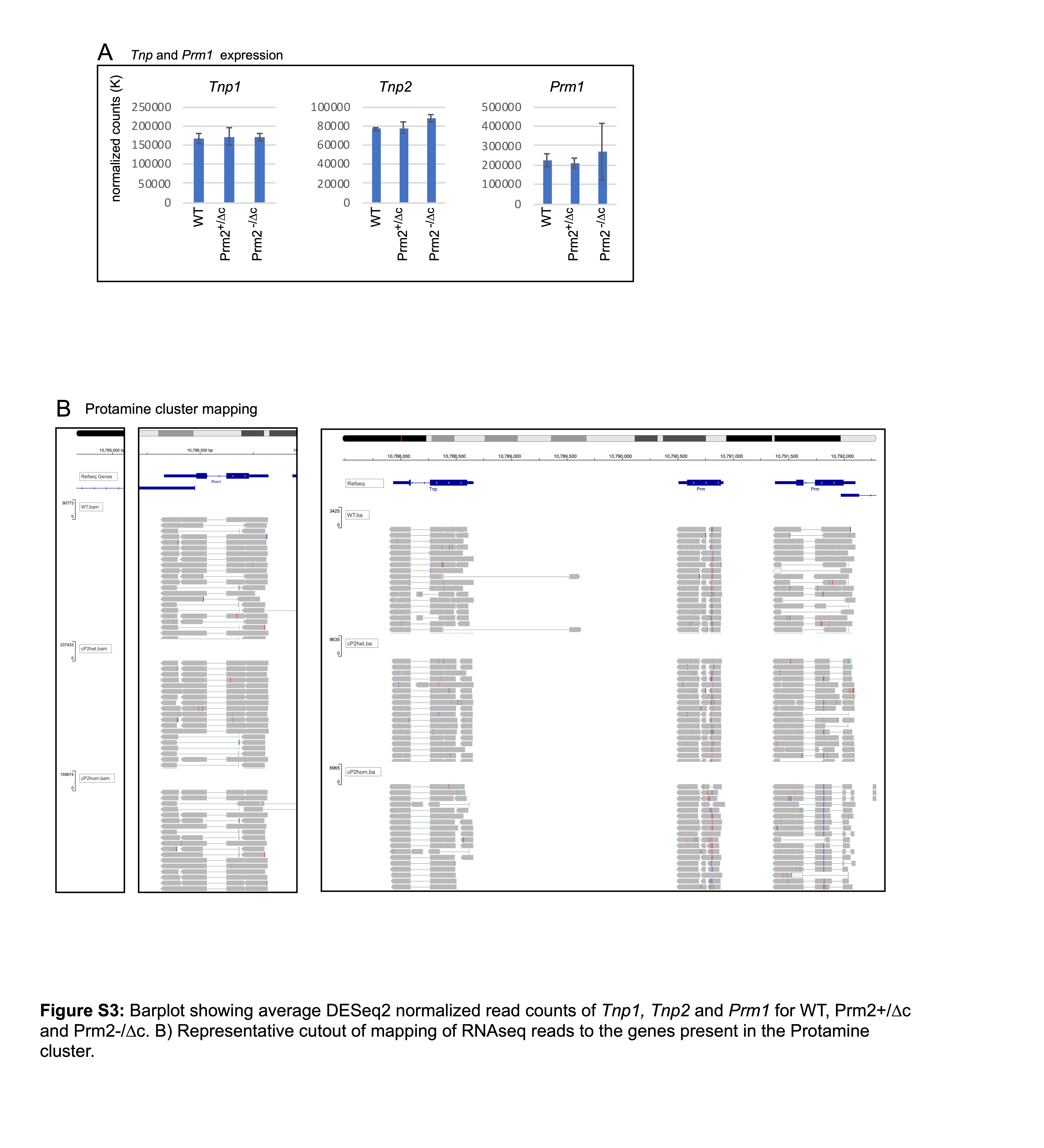

Supplement: S3 Fig — B) Representative cutout of mapping of RNAseq reads to the genes present in the Protamine cluster. (TIFF) [file pgen.1010272.s003.tiff]

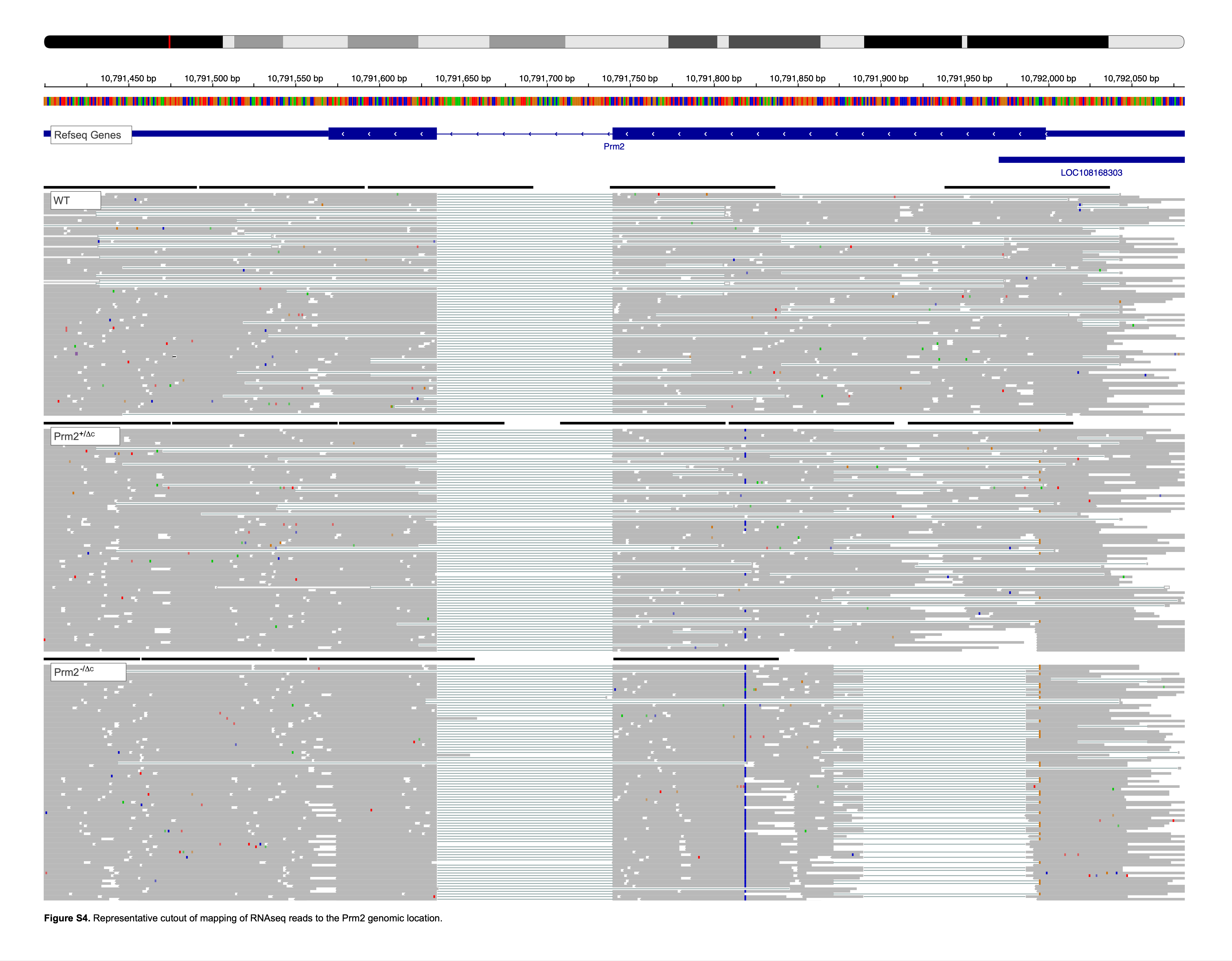

Supplement: S4 Fig — (TIFF) [file pgen.1010272.s004.tiff]

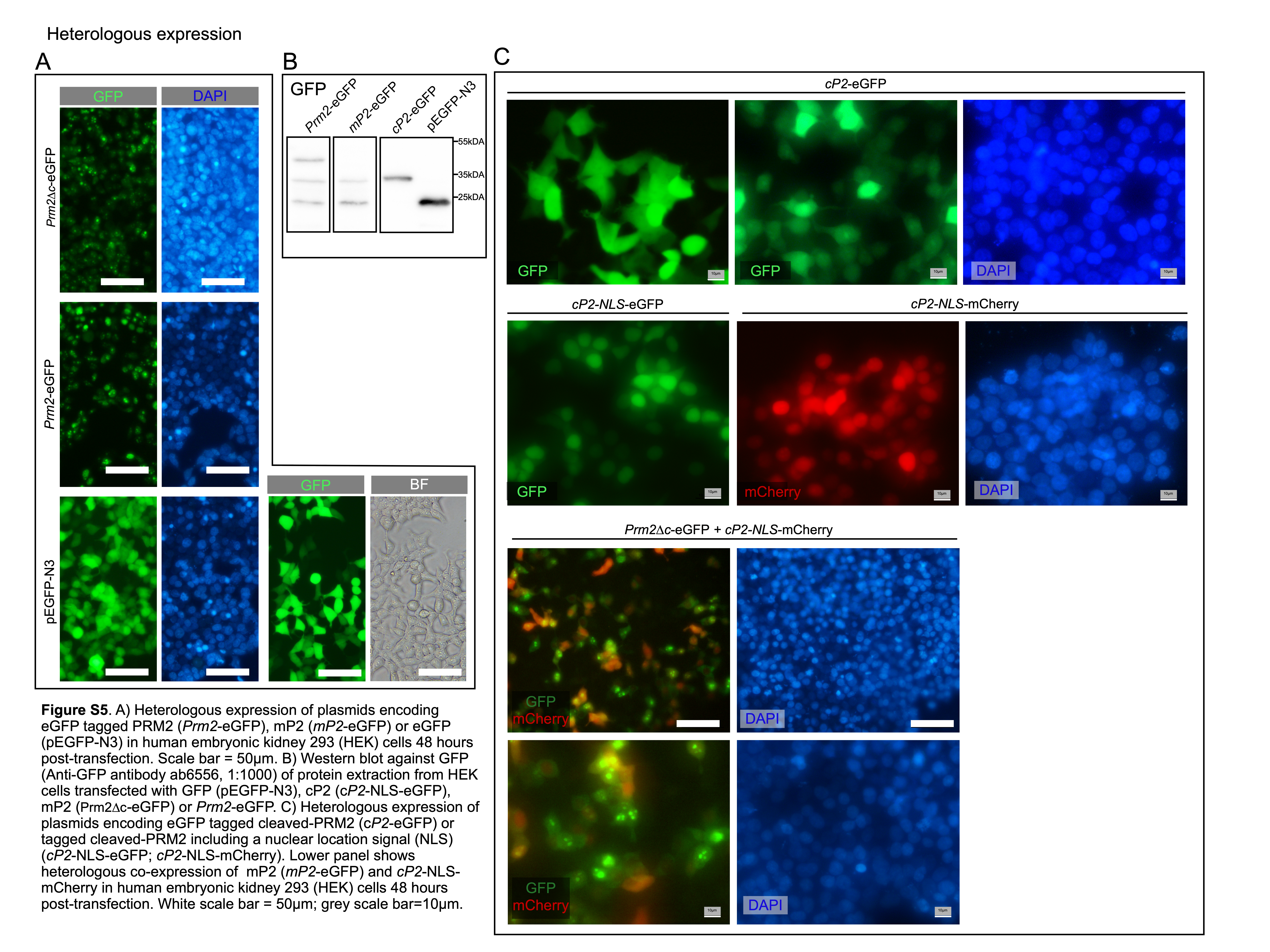

Supplement: S5 Fig — A) Heterologous expression of plasmids encoding eGFP tagged PRM2 (Prm2-eGFP), mP2 (mP2-eGFP) or eGFP (pEGFP-N3) in human embryonic kidney 293 (HEK) cells 48 hours post-transfection. Scale bar = 50μm. B) Western blot against GFP (Anti-GFP antibody ab6556, 1:1000) of protein extraction from HEK cells transfected with GFP (pEGFP-N3), cP2 (cP2-NLS-eGFP), mP2 (Prm2Δc-eGFP) or Prm2-eGFP. C) Heterologous expression of plasmids encoding eGFP tagged cleaved-PRM2 (cP2-eGFP) or tagged cleaved-PRM2 including a nuclear location signal (NLS) (cP2-NLS-eGFP; cP2-NLS-mCherry). Lower panel shows heterologous co-expression of mP2 (mP2-eGFP) and cP2-NLS-mCherry in human embryonic kidney 293 (HEK) cells 48 hours post-transfection. White scale bar = 50μm; grey scale bar = 10μm. (TIFF) [file pgen.1010272.s005.tiff]

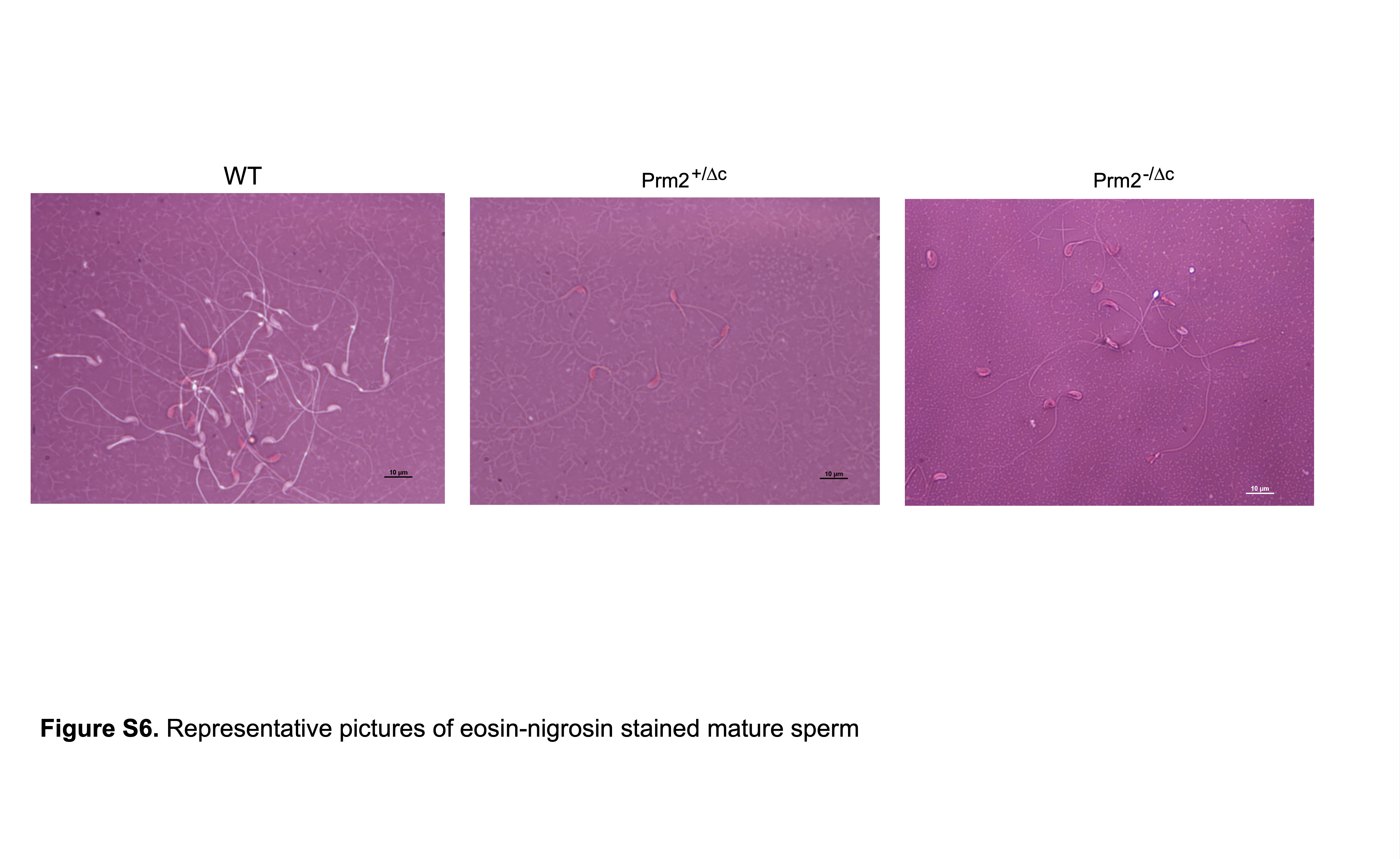

Supplement: S6 Fig — (TIFF) [file pgen.1010272.s006.tiff]

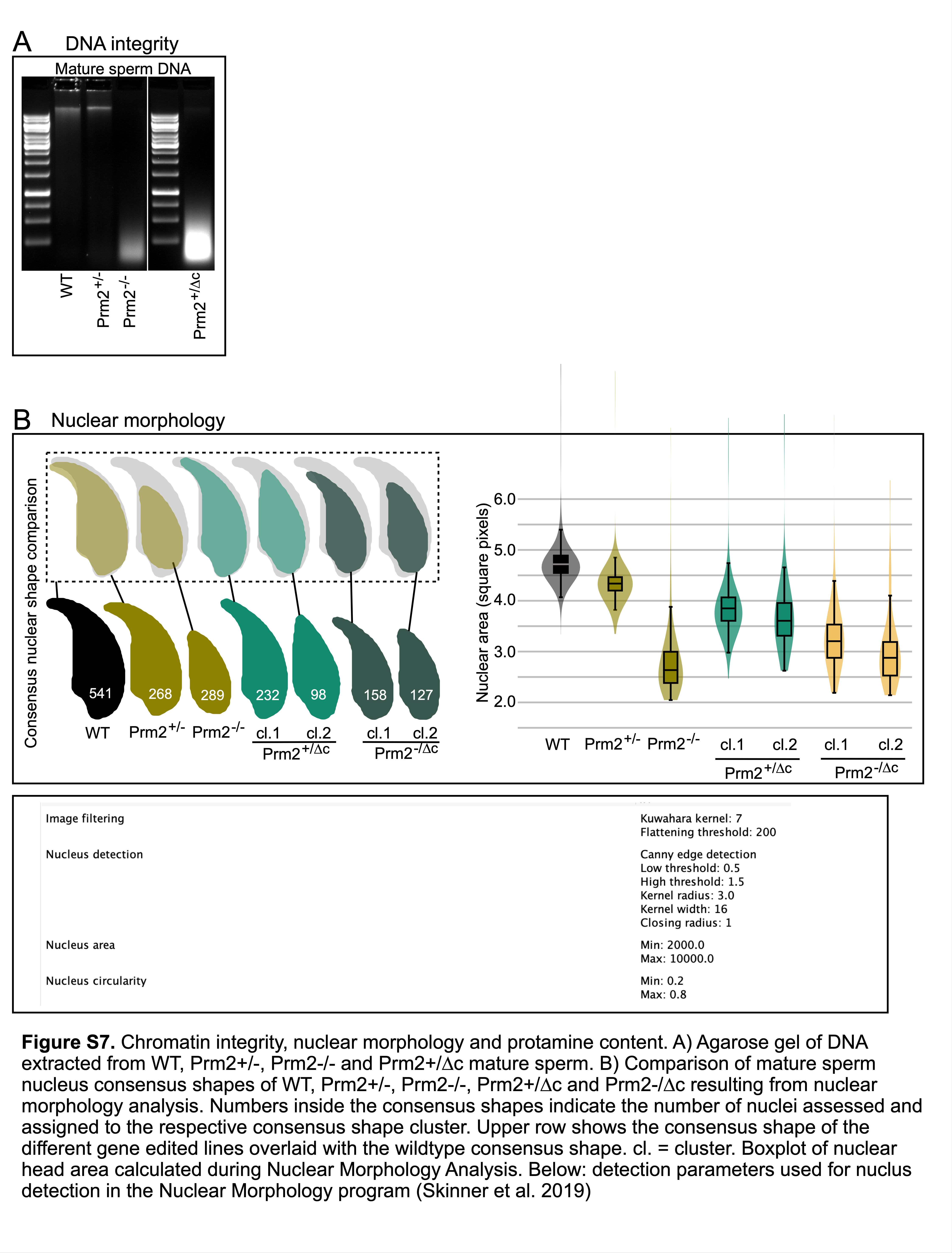

Supplement: S7 Fig — A) Agarose gel of DNA extracted from WT, Prm2+/-, Prm2-/- and Prm2+/Δc mature sperm. B) Comparison of mature sperm nucleus consensus shapes of WT, Prm2+/-, Prm2-/-, Prm2+/Δc and Prm2-/Δc resulting from nuclear morphology analysis. Numbers inside the consensus shapes indicate the number of nuclei assessed and assigned to the respective consensus shape cluster. Upper row shows the consensus shape of the different gene edited lines overlaid with the wildtype consensus shape. cl. = cluster. Boxplot of nuclear head area calculated during Nuclear Morphology Analysis. Below: detection parameters used for nuclus detection in the Nuclear Morphology program [33] (TIFF) [file pgen.1010272.s007.tiff]

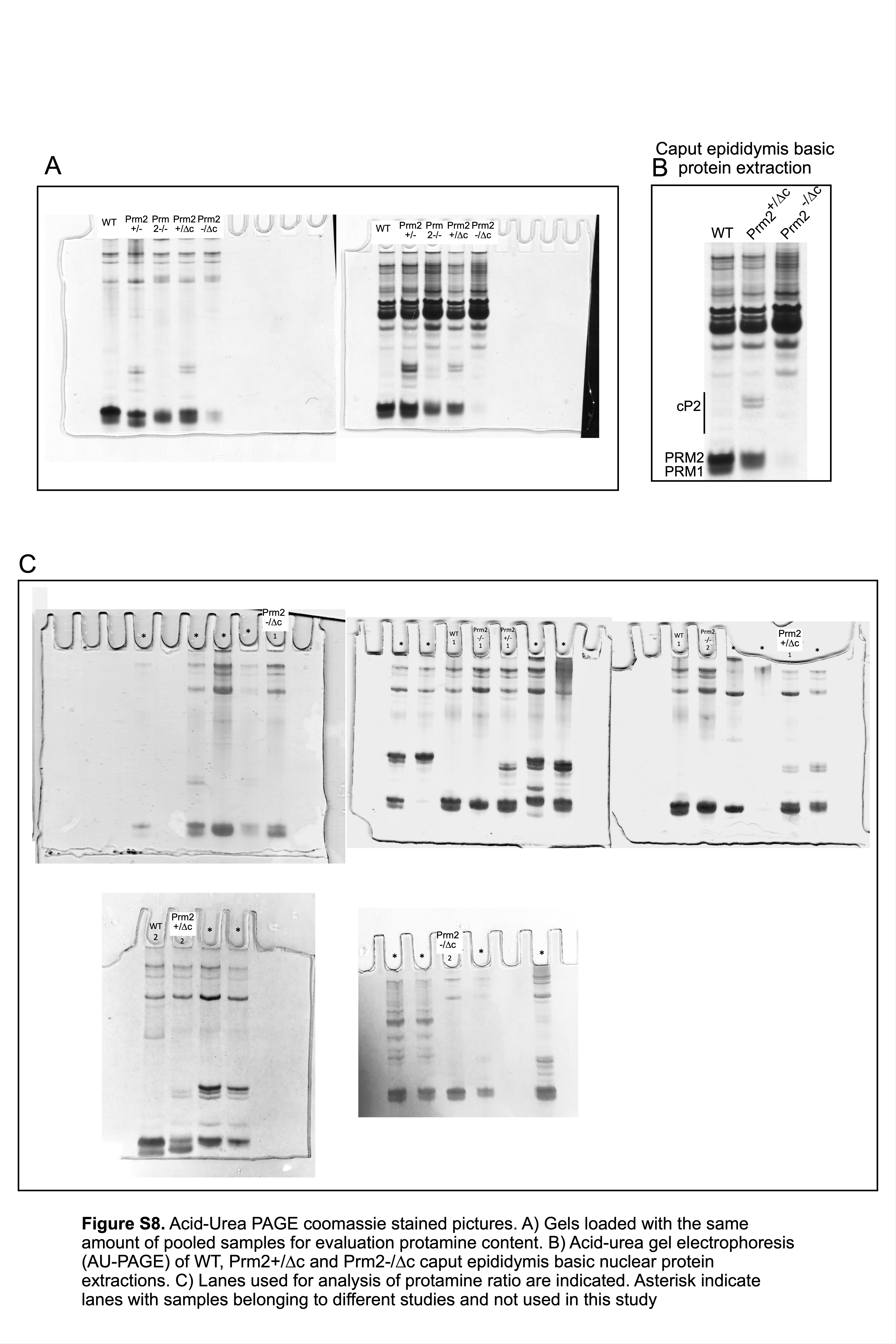

Supplement: S8 Fig — A) Gels loaded with the same amount of pooled samples for evaluation protamine content. B) Acid-urea gel electrophoresis (AU-PAGE) of WT, Prm2+/Δc and Prm2-/Δc caput epididymis basic nuclear protein extractions. C) Lanes used for analysis of protamine ratio are indicated. Asterisk indicate lanes with samples belonging to different studies and not used in this study. (TIFF) [file pgen.1010272.s008.tiff]

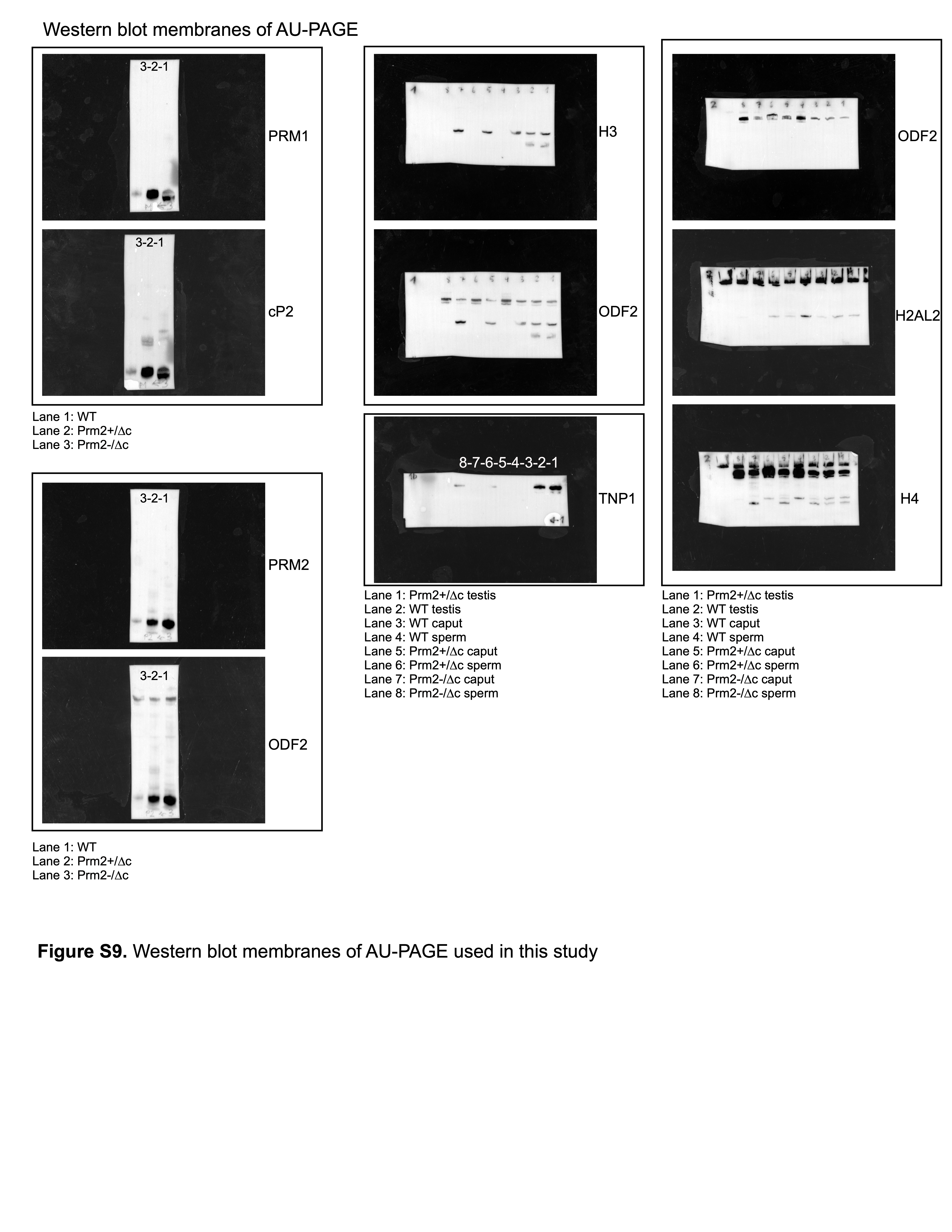

Supplement: S9 Fig — (TIFF) [file pgen.1010272.s009.tiff]

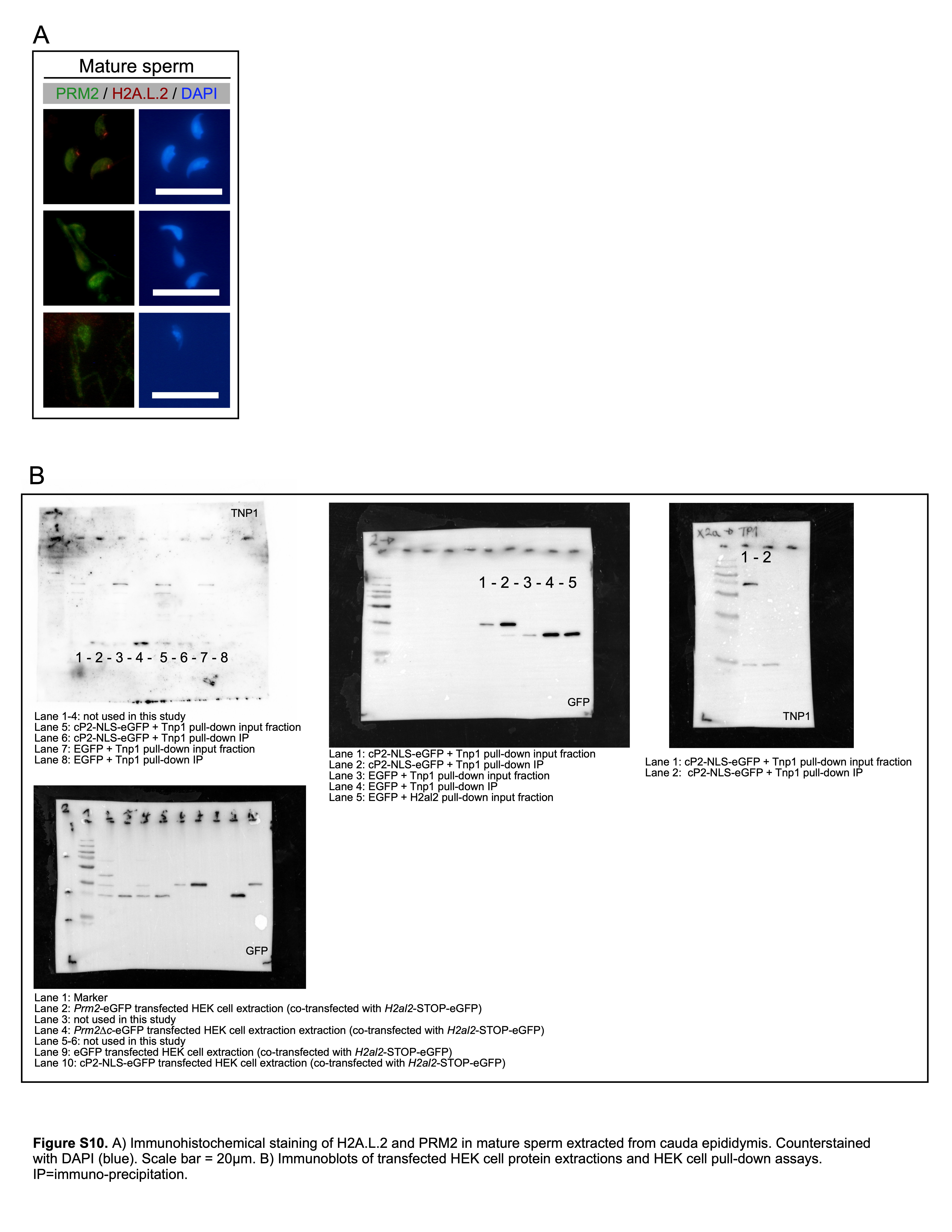

Supplement: S10 Fig — A) Immunohistochemical staining of H2A.L.2 and PRM2 in mature sperm extracted from cauda epididymis. Counterstained with DAPI (blue). Scale bar = 20μm. B) Immunoblots of transfected HEK cell protein extractions and HEK cell pull-down assays. IP = immuno-precipitation. (TIFF) [file pgen.1010272.s010.tiff]
